# High-Stability Ti_3_C_2_-QDs/ZnIn_2_S_4_/Ti(IV) Flower-like Heterojunction for Boosted Photocatalytic Hydrogen Evolution

**DOI:** 10.3390/nano12030542

**Published:** 2022-02-05

**Authors:** Liqin Yang, Zhihong Chen, Xin Wang, Mingliang Jin

**Affiliations:** 1 National Center for International Research on Green Optoelectronics, South China Academy of Advanced Optoelectronics, South China Normal University, Guangzhou 510006, China; yangliqin2019@126.com (L.Y.); wangxin@scnu.edu.cn (X.W.); 2 International Academy of Optoelectronics at Zhaoqing, South China Normal University, Zhaoqing 526000, China; chenzhihong1227@sina.com; 3 Key Laboratory for Water Quality and Conservation of the Pearl River Delta, Ministry of Education, Institute of Environmental Research at Greater Bay, Guangzhou University, Guangzhou 510006, China

**Keywords:** photocatalytic H_2_-evolution, ZnIn_2_S_4_ microspheres, Ti_3_C_2_ MXene quantum dots, amorphous Ti(IV), co-catalysts, synergistic effect

## Abstract

The practical application of photocatalytic H_2_-evolution is greatly limited by its sluggish charge separation, insufficient active sites, and stability of photocatalysts. Zero-dimensional (0D) Ti_3_C_2_ MXene quantum dots (MQDs) and amorphous Ti(IV) have been proven to be potential substitutes for noble co-catalyst to accelerate the separation of photogenerated electron-hole pairs and prevent the self-oxidation of photocatalysts, leading to better photocatalytic H_2_-evolution performance with long-term stability. In this study, amorphous Ti(IV) and MQDs co-catalysts were successfully deposited on ZnIn_2_S_4_ (ZIS) microspheres composed of ultra-thin nanosheets via a simple impregnation and self-assembly method (denoted as MQDs/ZIS/Ti(IV)). As expected, the optimal MQDs/ZIS/Ti(IV) sample exhibited a photocatalytic H_2_-evolution rate of 7.52 mmol·g^−1^·h^−1^ and excellent photostability without metallic Pt as the co-catalyst in the presence of Na_2_S/Na_2_SO_3_ as hole scavenger, about 16, 4.02 and 4.25 times higher than those of ZIS, ZIS/Ti(IV), and MQDs/ZIS, respectively. The significantly enhanced photocatalytic H_2_-evolution activity is attributed to the synergistic effect of the three-dimensional (3D) flower-like microsphere structure, the amorphous Ti(IV) hole co-catalyst, and a Schottky junction formed at the ZIS–MQDs interface, which offers more active sites, suppresses self-photocorrosion, and photo-generates the charge recombination of ZIS.

## 1. Introduction

Inspired by natural photosynthesis, the efficient conversion of solar energy into hydrogen via photocatalytic water splitting is regarded as a promising strategy to address the increasing environmental pollution and energy crisis because of its renewable solar energy sources and clean energy products [1,2,3]. In the past decades, numerous semiconductor-based photocatalysts, including metal-oxides [4,5,6,7], chalcogenides [8,9,10,11], organic-semiconductors [12,13,14], etc., have been developed and demonstrated to be candidate materials for photocatalytic H_2_-production. Among them, chalcogenide-based semiconductors are considered good candidates because of their suitable bandgap (most of them in the visible light response region) and band-edge positions [1,15,16,17]. In addition, most chalcogenide-based semiconductors have the advantages of controllable morphology, multi-dimensionality, and ultra-thin nanosheet structure, which opens up more possibilities for photocatalysis. In particular, three-dimensional (3D) ZnIn_2_S_4_ flower-like microspheres, benefiting from high light absorption efficiency, high specific surface area, unique surface properties, and plentiful attachment sites for doped substances, have shown promise for photocatalytic H_2_-evolution. However, pristine chalcogenides, similarly to most semiconductors, exhibit very low and even no photocatalytic H_2_-evolution activity because of the undesirable Gibbs free energy for proton reduction to H_2_ resulting from the lack of catalytic sites on their surfaces. In addition, when used by themselves, chalcogenides easily suffer from sluggish charge transfer, which leads to serious photo-corrosion and low photocatalytic activity, which in turn greatly obstructs their practical application in solar energy conversion to H_2_-energy [18,19,20,21]. Therefore, it is highly desirable to optimize the Gibbs free energy of chalcogenide-based photocatalysts to promote the reduction of proton to H_2_ and anti-corrosion properties for obtaining higher and more stable photocatalytic H_2_-evolution activity.

A new two-dimensional (2D) material discovered in 2011, Ti_3_C_2_ MXene, has attracted intensive and increasing attention in the fields of supercapacitors [22,23], electrochemical [24,25], batteries [26,27], and sensors [28,29] because of its excellent electrical conductivity, hydrophilicity, and mechanical and chemical stability [30,31,32,33]. Recently, Ti_3_C_2_ MXene has been regarded as a potential substitute material for Pt co-catalyst to facilitate the reduction of protons to H_2_ due to its Gibbs free energy for hydrogen adsorption and the fact that its Fermi level is close to zero, as is that of Pt [34,35,36,37,38]. For instance, Ran et al. first reported that the photocatalytic H_2_-evolution performance of CdS nanoparticles showed a significant enhancement by using Ti_3_C_2_ MXene nanoparticles as co-catalysts [39]. Xie et al. demonstrated that the incorporation of Ti_3_C_2_ MXene nanosheets into ZnIn_2_S_4_ nanosheets could quickly draw photogenerated electrons from the bulk phase and surface of ZnIn_2_S_4_ nanosheets to Ti_3_C_2_ MXene nanosheets by forming a Schottky junction. This is because Ti_3_C_2_ MXene nanosheets have near-zero Gibbs free energy for proton–H_2_ reactions, which can push the electrons on Ti_3_C_2_ MXene nanosheets to reduce proton, eventually leading to excellent Pt-free photocatalytic H_2_-evolution performance [40]. More recently, 2D Ti_3_C_2_ MXene nanosheets (MNSs) transformed into zero-dimensional (0D) Ti_3_C_2_ MXene quantum dots (MQDs) showed some unique physical and optical properties, which originated in deepening edge effects and quantum confinement effects, which could facilitate their application in boosting photocatalytic H_2_-evolution performance. Li et al. reported that the 0D MQDs-decorated g-C_3_N_4_ nanosheets (5111.8 μmol·g^−1^·h^−1^) exhibited approximately 10 times higher photocatalytic H_2_-evolution rates than 2D MNSs-decorated g-C_3_N_4_ nanosheets (524.3 μmol·g^−1^·h^−1^) due to the ability of the tiny particle size of the MQDs to increase the specific surface area, provide more edge active sites for H_2_-evolution reactions, and strengthen the contact between Ti_3_C_2_ MXene and g-C_3_N_4_ nanosheets [41].

Due to their unique quantum size structure, 0D QDs have better physical and optical properties and a higher photogenerated carrier separation rate than 2D or 3D structural materials. Among them, CuInS_2_ QDs [42], CdTe QDs [43] and MoC QDs [44] have been used to decorate the ZnIn_2_S_4_. Although the photocatalytic performance of modified ZnIn_2_S_4_ is better than that of pure ZnIn_2_S_4_, the photocatalytic activity of modified ZnIn_2_S_4_ is still poor, and it is still difficult to consider its practical application. MQDs also has excellent electrical conductivity, hydrophilicity, and mechanical and chemical stability, so it has attracted wide attention in recent years. However, although MQDs can accelerate the photogenerated electrons transfer, they can not act as hole co-catalysts to separate the photogenerated holes from the bulk phase and surface of chalcogenide-based photocatalysts; therefore, they can not effectively solve the photo-corrosion of chalcogenide-based photocatalysts. Previous investigations have demonstrated that the grafting of amorphous Ti(IV) on the surface of photocatalysts acts as a hole co-catalyst to effectively promote the photogenerated holes from the bulk phase and surface of photocatalysts to amorphous Ti(IV) [45,46,47]. In our recent study, we found that grafting amorphous Ti(IV) on the surface of flower-like ZnIn_2_S_4_ microspheres can not only accelerate the photogenerated charge separation, which results in remarkable enhanced photocatalytic H_2_-evolution performance, but also provide excellent long-time photo-stability [48]. Inspired by these studies, it is greatly anticipated that the simultaneous incorporation of amorphous Ti(IV) hole co-catalyst and MQDs electron co-catalyst on the surfaces of chalcogenides could accelerate the separation of photogenerated holes and electrons at the same time and optimize the Gibbs free energy for proton reduction to H_2_ without metallic Pt as the co-catalyst. This may create more opportunities for efficient and long-term photo-stability photocatalysis on chalcogenide-based photocatalysts, although this has not yet been reported.

In this study, a ternary composite photocatalyst that consisted of flower-like ZnIn_2_S_4_ (ZIS) microspheres with surficial deposition of amorphous Ti(IV) and MQDs co-catalysts (denoted as MQDs/ZIS/Ti(IV)) was first designed and synthesized via a simple impregnation and self-assembly method. As expected, all the MQDs/ZIS/Ti(IV) samples exhibited higher photocatalytic H_2_-evolution activity and greater stability than pure ZIS, ZIS/Ti(IV), and MQDs/ZIS without metallic Pt as co-catalyst in the presence of Na_2_S/Na_2_SO_3_ as the hole scavenger. In addition, the high-efficiency H_2_-evolution mechanism of MQDs/ZIS/Ti(IV) was investigated by steady-state photoluminescence spectroscopy (PL) and time-resolved fluorescence spectroscopy (TR-PL), and all the results suggested that the introduction of amorphous Ti(IV) and MQDs can significantly reduce the surface-photogenerated charge recombination rate of ZIS, thus notably improving photocatalytic H_2_-evolution performance. In particular, this study provides a new strategy for designing high-activity visible-light-driven photocatalysts and avoiding the use of precious metals as co-catalysts.

## 2. Experimental Section

### 2.1. Materials

All the reagents utilized were analytical grade and deionized (DI) water was used throughout the experiment. Ti_3_AlC_2_ powder, hydrofluoric acid, LiF powder were supplied from Sinopharm Chemical Reagent Co., Ltd., Shanghai, China. Indium chloride (InCl_3_·4H_2_O), zinc chloride (ZnCl_2_), sodium sulfide nonahydrate (Na_2_S·9H_2_O), thioacetamide (C_2_H_5_NS), sodium sulfite (Na_2_SO_3_), sodium sulphate (Na_2_SO_4_) and anhydrous ethanol (C_2_H_5_OH) were procured from Aladdin Reagent Company (Shanghai, China).

### 2.2. Sample Preparation

#### 2.2.1. Synthesis of ZIS Microspheres

In a typical synthesis, ZnCl_2_ (1 mmol), InCl_3_·4H_2_O (2 mmol), and C_2_H_5_NS (4 mmol) were dissolved in DI water (80 mL) with stirring treatment for 30 min, then transferred into a Teflon-lined autoclave and preserved at 80 °C for 12 h. The obtained yellow powder was purged with DI water several times and then dried at 60 °C for 10 h.

#### 2.2.2. Synthesis of MNSs and MQDs

A total of 2.0 g LiF powder was immersed in 40 mL of 9 M HCl with stirring for 30 min to obtain a stable suspension, and then 2.0 g Ti_3_AlC_2_ powder was slowly added to the suspension and stirred at 35 °C for 24 h. Next, the suspension was washed with DI water multiple times until the pH value of the supernatant dumped after centrifugation was 5, and the solid Ti_3_C_2_ powder was obtained. A total of 40 mL anhydrous ethanol was added to 1.0 g of Ti_3_C_2_ powder, ultrasonically treated for 1 h, then centrifuged at 10,000 rpm for 10 min to collect the suspended MNSs. A total of 20 mL DI water was added to the unexfoliated particles, placed in the cell-crushing apparatus for 1 h at 80% power, then centrifuged at 3500 rpm for 3 min to collect the suspended MNSs. The above steps were repeated several times to obtain more MNSs dispersions. Finally, MQDs were obtained by dispersing MNSs in the cell-crushing apparatus for 24 h at 80% power.

#### 2.2.3. Modification of ZIS by Amorphous Ti(IV) Co-Catalyst (ZIS/Ti(IV))

A simple impregnation method was utilized for the synthesis of amorphous Ti(IV)-modified ZIS microspheres. Our recent research found that 0.2 wt% amorphous Ti(IV)-modified ZIS microspheres showed the best H_2_ production performance. Therefore, 0.2 wt% amorphous Ti(IV)-modified ZIS microsphere composite photocatalyst was directly prepared. The specific preparation process was as follows: a 10 mL Ti(SO_4_)_2_ solution (0.2 g·L^−1^) was added into 1.0 g ZIS powder and stirred at 80 °C for 1 h. After the reaction, the ZIS/Ti(IV) powder was washed with DI water and then dried at 60 °C for 5 h in a vacuum oven.

#### 2.2.4. Modification of ZIS/Ti(IV) by MQDs Co-Catalyst (MQDs/ZIS/Ti(IV))

The 0.5 g ZIS/Ti(IV) powder and 5 mg·mL^−1^ MQDs solution with different volumes (1 mL, 2 mL, 3 mL, 4 mL) were dispersed in 40 mL DI water. The suspension was sonicated for 30 min, then stirred vigorously for 2 h before being washed with DI water several times to eliminate plethoric quantum dots and dried at 60 °C for 5 h in a vacuum oven to obtain MQDs_(x)_/ZIS/Ti(IV) composite photocatalyst (x = 1, 2, 3, 4, corresponding to the mass percentage of MQDs). For comparison, the MQDs_(x)_/ZIS and MNSs_(x)_/ZIS/Ti(IV) samples were prepared using the same method.

## 3. Results and Discussion

### 3.1. Characterization

**Figure 1:**
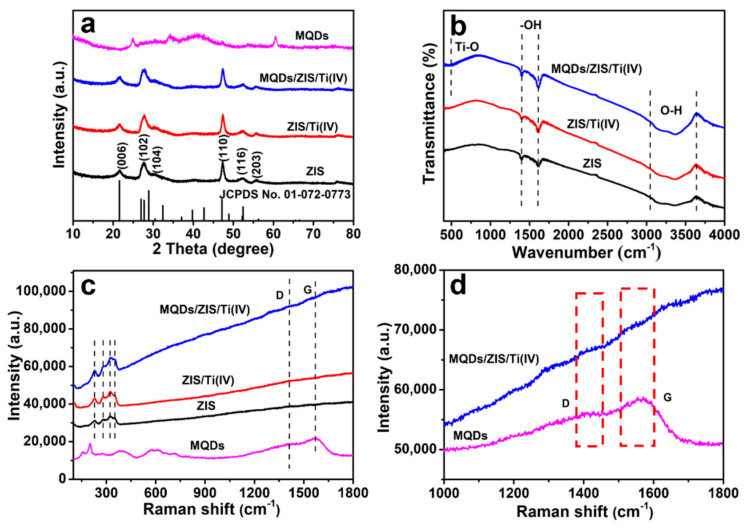
XRD patterns (**a**) of pure ZnIn_2_S_4_ (ZIS), ZnIn_2_S_4_/Ti(IV) (ZIS/Ti(IV)), Ti_3_C_2_ QDs/ZnIn_2_S_4_/Ti(IV) (MQDs/ZIS/Ti(IV)) and Ti_3_C_2_ QDs (MQDs) samples, FT-IR spectra (**b**) of ZIS, ZIS/Ti(IV) and MQDs/ZIS/Ti(IV) samples, Raman spectra (**c**) of ZIS, ZIS/Ti(IV), MQDs/ZIS/Ti(IV) and MQDs samples, enlarged portion (**d**) of MQDs/ZIS/Ti(IV) and MQDs Raman spectra.

Before the physical and chemical characterization of the as-prepared samples (the characterizations of the samples are described in the Supporting Information in detail), the optimal additive amount of amorphous Ti(IV) and MQDs co-catalysts were filtered by the univariate optimization method. The raw gas chromatography (GC) data (H_2_ peak area in GC pattern vs. time) and quantitative analysis of the photocatalytic H_2_ production results are listed in Table S1. Therefore, in the following section, ZIS/Ti(IV) and MQDs/ZIS represent the ZIS modified with optimal amorphous Ti(IV) and MQDs co-catalysts, respectively, with the best photocatalytic H_2_-production activity. Furthermore, MQDs/ZIS/Ti(IV) represents the sample co-modified by amorphous Ti(IV) and MQDsco-catalysts with the best photocatalytic H_2_-production activity. The crystal structure of the as-prepared samples was characterized by X-ray powder diffraction (XRD). As shown in Figure 1a, pristine ZIS exhibited diffraction peaks typical of the hexagonal ZIS phase (JCPDS No. 01-072-0773) at 21.5, 27.7, 30.6, 47.3, 52.4, and 56.1°, which were indexed to the (006), (102), (104), (110), (116) and (203) crystal planes of the hexagonal ZIS, respectively [48], and the XRD pattern of the MQDs was consistent with those reported in previous studies [38,41]. With the deposition of amorphous Ti(IV) and MQDs co-catalysts, ZIS/Ti(IV) and MQDs/ZIS/Ti(IV) also showed the typical hexagonal ZIS phase, which demonstrates that the co-modification of amorphous Ti(IV) and MQDs co-catalysts did not change the crystal structure of ZIS. However, no typical diffraction peaks for amorphous Ti(IV) and MQDs were observed in the MQDs/ZIS/Ti(IV), which was ascribed to the low amount and high dispersion of amorphous Ti(IV) and MQDs.

Fourier-transform infrared (FT-IR) spectroscopy was employed to confirm the successful synthesis of the MQDs/ZIS/Ti(IV) composites. As shown in Figure 1b, ZIS and ZIS/Ti(IV) showed similar FT-IR spectra with peaks at 1400 and 1620 cm^−1^ corresponding to the absorbed H_2_O molecules on the surface of ZIS, and peaks at 3000–3600 cm^−1^ correspond to the stretching vibration models of O-H in absorbed H_2_O molecules [49,50,51,52]. Further observation indicated that the intensity of these peaks for ZIS/Ti(IV) was higher than that of ZIS due to the hydrophilic ability of amorphous Ti(IV), implying that amorphous Ti(IV) was successfully deposited on the surface of ZIS by the impregnation method. Compared with ZIS and ZIS/Ti(IV), a new absorption peak at 503 cm^−1^, corresponding to the Ti-O stretching vibration mode of the MQDs, and a higher intensity absorption peak of 3000–3600 cm^−1^, corresponding to the OH-enriched surfaces of the MQDs, were observed in the FT-IR spectra of MQDs/ZIS/Ti(IV), demonstrating that MQDs were successfully deposited on ZIS/Ti(IV) composites by the self-assembly method [53]. Moreover, the Raman spectrum was carried out to confirm the successful deposition of amorphous Ti(IV) and MQDs on the ZIS surface, and the results are shown in Figure 1c. The pure ZIS exhibited five characterized Raman peaks at 107, 228, 285, 324, and 352 cm^−1^, corresponding to the layered structure, longitudinal optical mode (LO_1_), transverse optical mode (TO_2_), longitudinal optical mode (LO_2_), and A_1g_ mode of ZIS, respectively. For ZIS/Ti(IV), all the primary peaks of ZIS were observed and no typical peaks were observed for amorphous Ti(IV), owing to the low amount and high dispersion of amorphous Ti(IV). Compared with ZIS/Ti(IV), MQDs/ZIS/Ti(IV) exhibited two new peaks at 1409 and 1570 cm^−1^, corresponding to the D and G bands of the MQDs generated during the chemical etching process. As shown in Figure 1d, the intensity ratios of the D to G bands (I_D_/I_G_) were 0.94 for the MQDs/ZIS/Ti(IV) composites, which was higher than the pure MQDs (I_D_/I_G_ = 0.86), indicating that MQDs were successfully integrated into the lattice of ZIS/Ti(IV) and that there was intensive electron transfer between the ZIS and MQDs electron co-catalyst [54].

**Figure 2:**
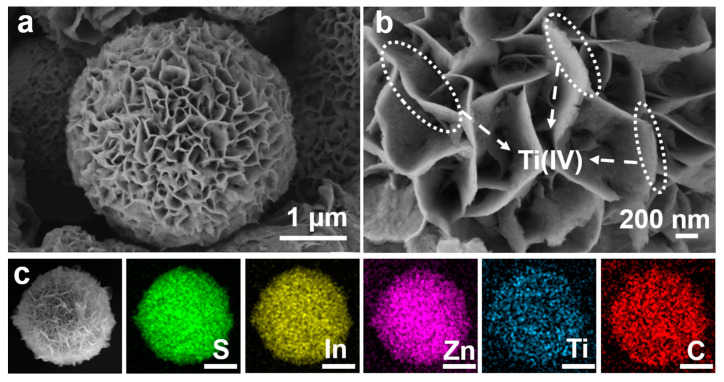
SEM images of MQDs/ZIS/Ti(IV) (**a**,**b**); corresponding elemental mappings of S, In, Zn, Ti, and C (**c**).

Scanning electron microscopy (SEM) was applied to obtain the morphological structure of the as-prepared samples. As displayed in Figure S1, the pristine ZIS showed a flower-like microsphere structure with a diameter of 3–6 µm composed of self-assembly ZIS nanosheets, which is conducive to light absorption and photogenerated carrier transportation. After being deposited with amorphous Ti(IV), no obvious changes in the flower-like microsphere structure were observed for ZIS/Ti(IV) but obvious additional folds and the uniform distribution of Ti elements were observed throughout the ZIS microspheres (Figure S2), implying that the impregnation method can not only ensure the uniform deposition of amorphous Ti(IV), but also does not change the microsphere structure of ZIS. As shown in Figure 2a,b, MQDs/ZIS/Ti(IV) displayed the same flower-like microsphere structure as ZIS/Ti(IV), suggesting that the further deposition of MQDs on the surface of ZIS/Ti(IV) by the self-assembly method did not destroy its flower-like microsphere structure. In addition, no typical quantum dot structure was observed in the MQDs in MQDs/ZIS/Ti(IV), which was ascribed to the tiny particle size of the MQDs, beyond the range of SEM observation. It can be observed that ZIS exhibited a specific surface area of 98.30 m^2^·g^−1^ (Figure S3). After being modified with amorphous Ti(IV) and MQDs, the specific surface area of MQDs/ZIS/Ti(IV) increased to 117.91 m^2^·g^−1^, indicating that the deposition of amorphous Ti(IV) and MQDs can not only guarantee the microspheres structure of ZIS, but also provide more active sites for photocatalytic H_2_ evolution, which is pivotal in photocatalytic reaction. SEM elemental mappings of the MQDs/ZIS/Ti(IV) microspheres reveal that S, In, Zn, and Ti, along with an additional C, are evenly distributed on the whole MQDs/ZIS/Ti(IV) (Figure 2c), suggesting that MQDs are uniformly deposited on the surface of ZIS/Ti(IV), which can be further confirmed by the increase in the Ti content of MQDs/ZIS/Ti(IV) compared with the ICP-MS results of ZIS/Ti(IV) (Table S2).

**Figure 3:**
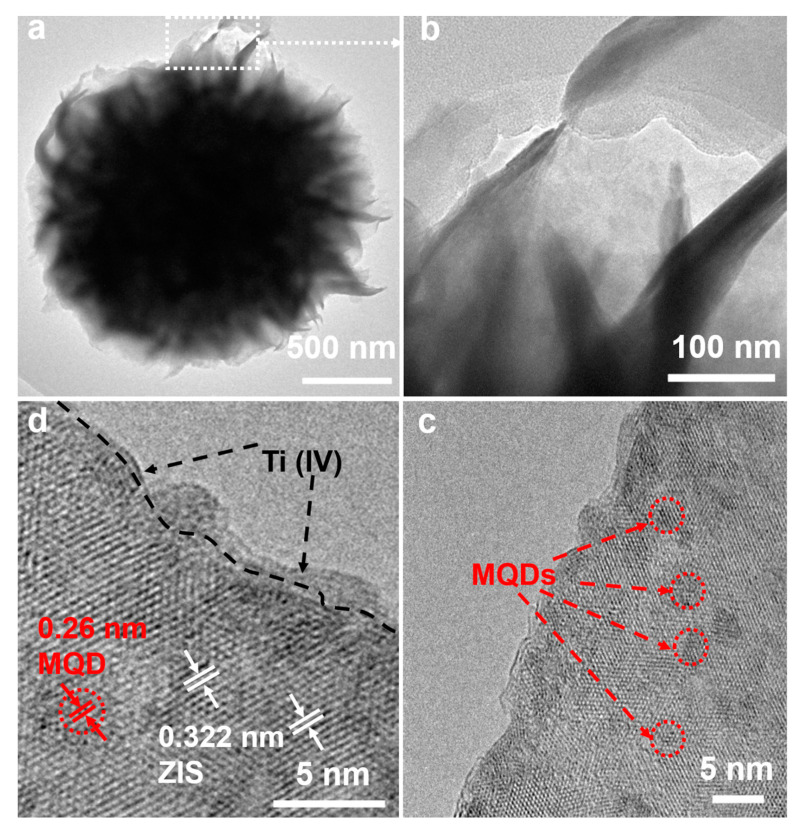
TEM (**a**,**b**) and HRTEM (**c**,**d**) images of MQDs/ZIS/Ti(IV).

The transmission electron microscopy (TEM) results further confirmed the successful construction of MQDs/ZIS/Ti(IV) and their interfacial contact. The MQDs prepared by the cell fragmentation method showed lattice fringe interplanar spacing of 0.26 nm, corresponding to the (110) plane of Ti_3_C_2_ (Figure S4b). According to the TEM image of the MQDs (Figure S4a), the diameter distribution of the MQDs shows a clear quantum dot structure with a size of 2–5 nm, and the majority of MQDs were 3.5 nm in diameter (Figure S5). MQDs/ZIS/Ti(IV) displayed a nanosheet assembly structure, which is in good agreement with the results of the SEM (Figure 3a,b). As shown in Figure 3c,d, the lattice fringe interplanar spacing of 0.322 nm corresponded to the (102) plane of the hexagonal ZIS, 0.26 nm corresponded to the (110) plane of the MQDs, and an amorphous Ti(IV) nanocluster was observed at the edge of the MQDs/ZIS/Ti(IV) sample. These results indicate the successful co-deposition and close contact between amorphous Ti(IV) and MQDs on the surface of flower-like ZIS microspheres, which is conducive to the transfer and separation of charge carriers between ZIS, amorphous Ti(IV), and MQDs co-catalysts, thus improving photocatalytic activity.

**Figure 4:**
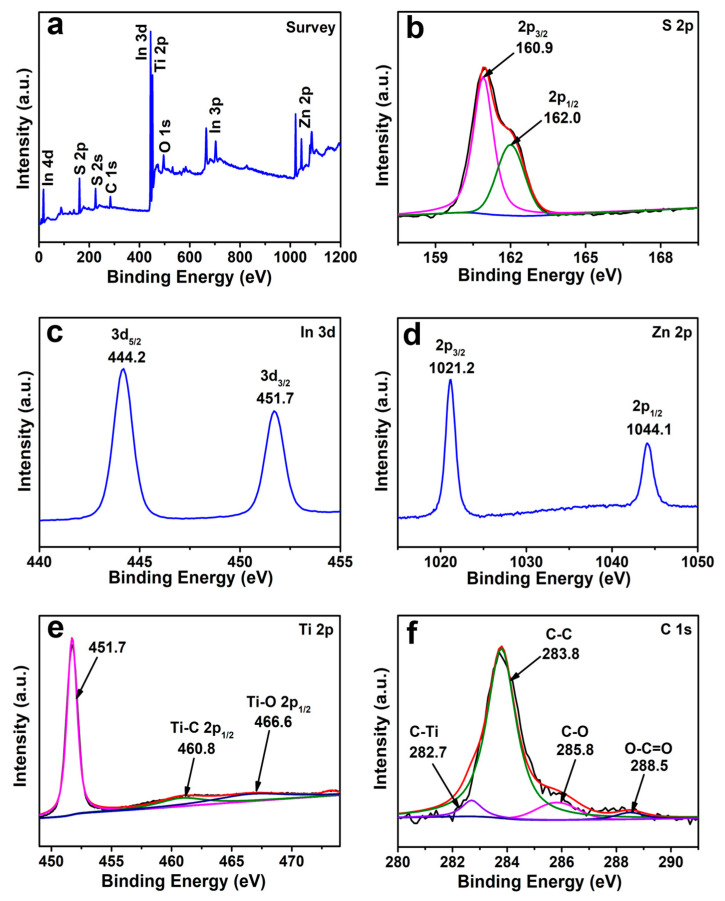
XPS spectra of MQDs/ZIS/Ti(IV): survey (**a**), S 2p (**b**), In 3d (**c**), Zn 2p (**d**), Ti 2p (**e**), C 1s (**f**).

Moreover, the chemical state and surface elemental composition of MQDs/ZIS/Ti(IV) were further studied by X-ray photoelectron spectroscopy (XPS). As shown in Figure 4a, the survey XPS spectrum of MQDs/ZIS/Ti(IV) was consistent with the corresponding EDX element mappings, and the O element in the sample belonged to oxygen originating from absorbed water. The S 2p high-resolution XPS spectrum exhibited two peaks, at 160.8 eV for S 2p_3/2_ and at 161.8 eV for S 2p_1/2_ (Figure 4b). The In spectrum (two bands at 444.1 eV for In 3d_5/2_ and 451.7 eV for In 3d_3/2_) indicated that In was in the +3 state (Figure 4c). The Zn spectrum (Figure 4d) revealed peaks at 1021.1 eV and 1044.1 eV, corresponding to the Zn 2p_3/2_ and Zn 2p_1/2_, respectively, indicating that Zn was in the +2 state. Figure S6a–c shows the slight shift toward higher binding energy for the peaks of S 2p, In 3d, and Zn 2p in ZIS/Ti(IV) in comparison with that of ZIS, which was due to the electron contribution of ZIS to the amorphous Ti(IV) nanoclusters and the strong interaction between the components. Meanwhile, ZIS/Ti(IV) exhibited significant Ti 2p XPS peaks (the band at 451.7 eV for the superposition of In 3d and Ti 2p, two bands at 461.4 eV for Ti 2p_3/2_ and 467.8 eV for Ti 2p_1/2_), indicating the presence of Ti^4+^ (Figure S6d), which further confirms the successful adhesion of amorphous Ti(IV) to the ZIS nanosheets [40,55]. The Ti 2p XPS spectrum of MQDs/ZIS/Ti(IV) can be compartmentalized into three kinds of contributions, located at 451.7, 460.8, and 466.6 eV, which can be assigned to the superposition of In 3d and Ti 2p, Ti-C 2p_1/2_, and Ti-O 2p_1/2_, respectively (Figure 4e). In addition, the C 1s XPS spectrum can also be partitioned into four components, located at 282.7, 283.8, 285.8, and 288.5 eV, corresponding to C-Ti, C-C, C-O, and O-C=O, respectively (Figure 4f). These results further demonstrate the successful synthesis of the MQDs/ZIS/Ti(IV) composite photocatalyst [56].

**Scheme 1:**
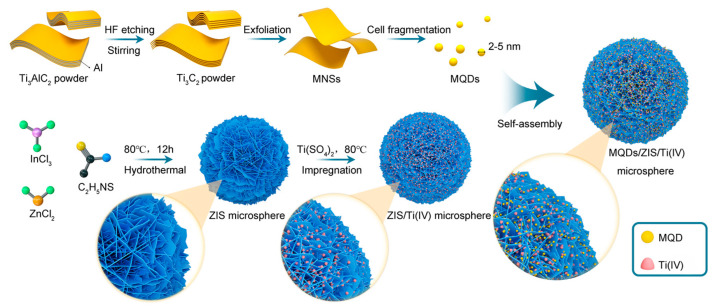
Schematic illustration of the preparation of MQDs/ZIS/Ti(IV) composite photocatalyst.

Based on the above analysis, MQDs/ZIS/Ti(IV) was successfully synthesized via a simple impregnation and self-assembly method, and the synthesis process is shown in Scheme 1. Firstly, MQDs and flower-like ZIS microspheres were prepared through wet chemical etching-ultrasonic exfoliation-cell fragmentation and the hydrothermal method, respectively. Secondly, amorphous Ti(IV) was deposited on the surface of the flower-like ZIS microsphere nanosheets by the impregnation method to obtain ZIS/Ti(IV). Finally, flower-like ZIS/Ti(IV) microspheres were used to support the in situ growth of MQDs by the self-assembly method to obtain MQDs/ZIS/Ti(IV).

### 3.2. Photocatalytic Properties

**Figure 5:**
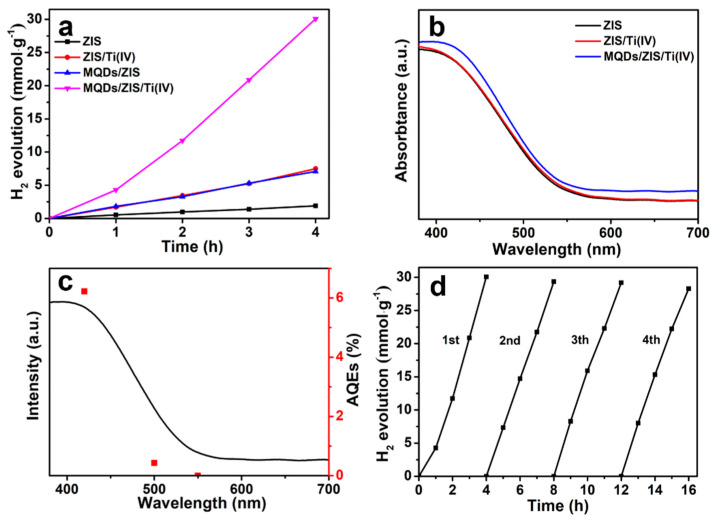
The photocatalytic H_2_-evolution of ZIS, ZIS/Ti(IV), MQDs/ZIS and MQDs/ZIS/Ti(IV) samples under simulated sunlight irradiation (AM.1.5) (**a**); UV–vis diffuse reflection spectra of ZIS, ZIS/Ti(IV) and MQDs/ZIS/Ti(IV) samples (**b**); wavelength-dependent AQEs of MQDs/ZIS/Ti(IV) (**c**); the stability test for four cycling H_2_ evolution of MQDs/ZIS/Ti(IV) under simulated sunlight irradiation (AM.1.5) (**d**).

The photocatalytic H_2_-evolution activities of the as-prepared samples were evaluated without metallic Pt as co-catalyst in the presence of Na_2_S/Na_2_SO_3_ as hole scavenger (photocatalytic H_2_-production experimental details are described in the Supporting Information) and the results are shown in Figure 5 and Figure S7. MQDs/ZIS/Ti(IV) exhibited remarkably higher H_2_-evolution activity (7.52 mmol·g^−1^·h^−1^) than ZIS (0.47 mmol·g^−1^·h^−1^), ZIS/Ti(IV) (1.87 mmol·g^−1^·h^−1^), MQDs/ZIS (1.77 mmol·g^−1^·h^−1^), and the sum of ZIS/Ti(IV) and MQDs/ZIS (3.64 mmol·g^−1^·h^−1^), indicating that there was a synergistic effect between amorphous Ti(IV) grafting and MQDs deposition, which can more effectively improve the photocatalytic H_2_-evolution activity of ZIS than the modification of amorphous Ti(IV) and MQD co-catalysts alone. The GC diagram of the best sample (MQDs/ZIS/Ti(IV)) at each time (1, 2, 3 and 4 h) is shown in Figure S8a–d. As shown in Table S3, compared with other works on ZIS photocatalysts integrated with quantum dots, it is clearer that the co-modification of ZIS by amorphous Ti(IV) and MQDs is an effective way to improve the photocatalytic activity of ZIS. Moreover, it can be seen from Table S4 that the H_2_-evolution activity of the MQDs/ZIS/Ti(IV) sample was also higher than that of the MNSs/ZIS/Ti(IV) sample (ZIS co-modified by amorphous Ti(IV) and MNSs co-catalysts, for which the SEM image of the MNSs is shown in Figure S9), which was attributed to the fact that MQDs can provide more absorption sites for H^+^ and reduction sites for H_2_-evolution than MNSs. Compared with MNSs, the FT-IR spectra of the MQDs exhibited stronger peaks at 3180.9, 1483.4, 1251.9, and 595.4 cm^−1^, corresponding to the stretching vibration modes of the -OH, C = O, C-F, and Ti-O groups (Figure S10), respectively, indicating that the terminating groups of MQDs possessed strong hydrophilicity, which is beneficial in the contact between MQDs and water, improving photocatalytic activity [53,57]. In addition, the MNSs were coated on the surfaces of the ZIS microspheres rather than deposited on their sheets (Figure S11), resulting in a decrease in light absorption (Figure S12) and surface area for ZIS (Figure S3), which is harmful to photocatalytic H_2_-evolution reactions. The optical absorption capacities of the as-prepared samples were studied by UV−vis diffuse reflectance spectroscopy (DRS). Figure 5b shows the UV-vis spectra of ZIS, ZIS/Ti(IV), and MQDs/ZIS/Ti(IV). The pure ZIS showed strong and broad visible light absorption in the range of 400–550 nm. After being deposited with amorphous Ti(IV) co-catalyst, ZIS/Ti(IV) showed a similar absorption curve to pure ZIS, resulting from the low amount and high dispersion of amorphous Ti(IV). After being further deposited with MQDs co-catalyst, the light absorption of MQDs/ZIS/Ti(IV) in the visible light region increased slightly and the absorption edge redshifted, owing to the strong long-wavelength absorption property of MQDs (Figure S12), which is beneficial for the effective utilization of solar energy.

To further confirm that the process of photocatalytic H_2_-production can be efficiently driven by the visible light absorption of ZIS, wavelength-dependent H_2_-production experiments were also carried out under different monochromatic lights. As shown in Figure 5c, MQDs/ZIS/Ti(IV) exhibited the best photocatalytic activity at 420 nm, which corresponded well to the results of the DRS spectrum, indicating that the photocatalytic reduction process is primarily driven by light-induced of electrons in the MQDs/ZIS/Ti(IV) [3]. The calculated apparent quantum efficiencies (AQEs) under 420, 500, and 550 nm monochromatic light irradiation were 6.22, 0.43, and 0%, respectively, confirming that the co-modification of amorphous Ti(IV) and MQDs co-catalysts is a more feasible strategy to enhance the photocatalytic activity of ZIS, especially in the long-wavelength region.

The stability of photocatalyst is an important index for its large-scale application, and MQDs/ZIS/Ti(IV) has been continuously tested for four cycles of photocatalytic H_2_ evolution under identical reaction conditions. As shown in Figure 5d, the change in the photocatalytic H_2_ production rate of MQDs/ZIS/Ti(IV) was almost negligible after four cycles, suggesting that the composite photocatalyst was sufficiently photo-stable for photocatalytic H_2_ evolution. The raw H_2_ peak area in GC pattern vs. time data and quantitative analysis of the photocatalytic H_2_ production results are listed in Table S5. Furthermore, the MQDs/ZIS/Ti(IV) after the photocatalytic reaction was characterized by XRD and XPS to verify the stability of the crystal structure and chemical states, respectively. The XRD patterns (Figure S13) and XPS spectrum (Figure S14) of MQDs/ZIS/Ti(IV) did not show any obvious changes before or after photocatalytic reaction, indicating that MQDs/ZIS/Ti(IV) possesses excellent stability for photocatalytic H_2_-evolution.

**Figure 6:**
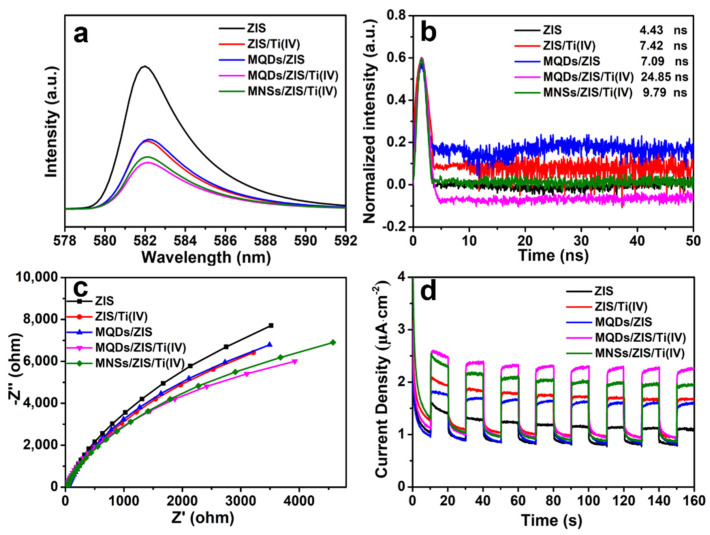
PL spectra (**a**), TR-PL spectra (**b**); EIS (**c**) and transient photocurrent response (**d**) of ZIS, ZIS/Ti(IV), MQDs/ZIS, MQDs/ZIS/Ti(IV), and MNSs/ZIS/Ti(IV) samples.

According to the photocatalytic H_2_-evolution tests, the co-modification of amorphous Ti(IV) and MQDs co-catalysts on the ZIS surface separates the photogenerated charge more effectively than modifying amorphous Ti(IV) or MQDs co-catalysts alone, suggesting a synergistic effect between amorphous Ti(IV) and MQDs co-catalysts for charge transfer, which was further confirmed by PL, TR-PL, EIS, and transient photocurrent response tests. It is well known that PL irradiation energy results from the recombination of photogenerated electron-hole pairs; hence, a higher photoluminescence intensity means a higher recombination rate of photogenerated carriers [13]. As shown in Figure 6a, compared with ZIS, ZIS/Ti(IV), MQDs/ZIS, MQDs/ZIS/Ti(IV), and MNSs/ZIS/Ti(IV) showed lower PL intensity, and MQDs/ZIS/Ti(IV) displayed the lowest PL intensity, indicating that the synergistic effect of the amorphous Ti(IV) and MQDs co-catalysts accelerated the separation and transportation of the photogenerated electron-hole pairs more efficiently. MNSs/ZIS/Ti(IV) showed a higher PL peak intensity than MQDs/ZIS/Ti(IV), owing to weak contact between the ZIS and MNSs, leading to weaker carrier transport, which is consistent with the results of the SEM.

To further quantitatively study the carrier transfer dynamics, TR-PL decay spectra were performed. All the TR-PL decay curves were fitted by bi-exponential function $y=A_{1}\exp\left(-\frac{t}{\tau_{1}}\right)+A_{2}\exp\left(-\frac{t}{\tau_{2}}\right)$, and the corresponding effective lifetimes (τ_eff_) were extracted by using the formula: $\tau_{\mathrm{eff}}=\left(A_{1}\tau_{1}+A_{2}\tau_{2}\right)/\left(A_{1}+A_{2}\right)$. The detailed fitting parameters are listed in Table S. The τ_eff_ of ZIS, ZIS/Ti(IV), MQDs/ZIS, MQDs/ZIS/Ti(IV), and MNSs/ZIS/Ti(IV) are 4.43, 7.42, 7.09, 24.85, and 9.79 ns, respectively (Figure 6b). The carrier lifetime of MNSs/ZIS/Ti(IV) was shorter than that of MQDs/ZIS/Ti(IV), implying that the electron transfer between MQDs and ZIS was more efficient than that between MNSs and ZIS, indicating that the MQDs had better contact with ZIS than MNSs. The carrier lifetime of ZIS/Ti(IV), MQDs/ZIS and MQDs/ZIS/Ti(IV) was longer than that of the pure ZIS; MQDs/ZIS/Ti(IV) exhibited the longest carrier lifetime, demonstrating that decorated amorphous Ti(IV) and MQDs co-catalysts can act as hole and electron acceptors, respectively, to enhance carrier dissociation and improve carrier lifetime.

**Figure 7:**
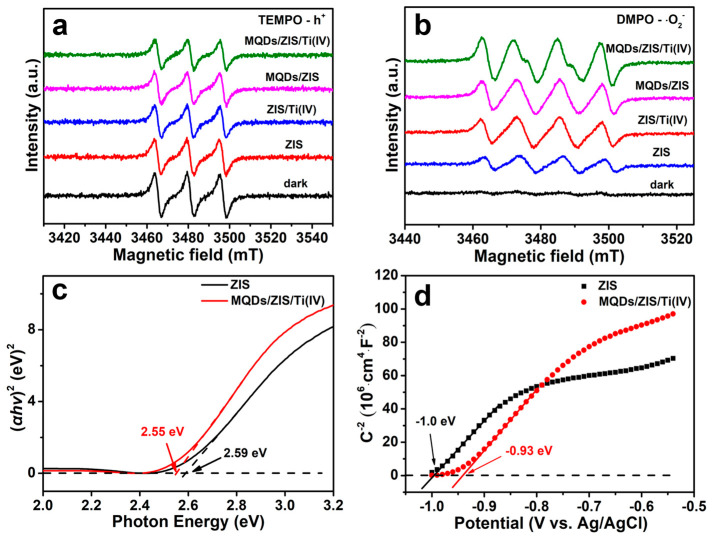
EPR spectra of TEMPO-h^+^ (**a**) and DMPO-·O_2_^−^ (**b**) of ZIS, ZIS/Ti(IV), MQDs/ZIS and MQDs/ZIS/Ti(IV) samples; (*αhv*)^2^ vs. photon energy plot from UV–vis DRS analysis (**c**) and Mott–Schottky plot (**d**) of ZIS and MQDs/ZIS/Ti(IV) samples.

The EIS was also used to study the interfacial charge transfer behavior of the as-prepared samples (the photoelectrochemical measurement details are described in the Supporting Information). As shown in Figure 6c, the arc radius of ZIS/Ti(IV) and MQDs/ZIS was smaller than that of ZIS and the arc radius of MQDs/ZIS/Ti(IV) was the smallest of all the samples, indicating that the MQDs/ZIS/Ti(IV) possessed the fastest interfacial charge transfer rate. As expected, the arc radius of MNSs/ZIS/Ti(IV) was larger than that of MQDs/ZIS/Ti(IV). At the same time, the transient photocurrent response of the as-prepared samples was prompted by several on/off cycles of light, and MQDs/ZIS/Ti(IV) showed the highest transient photocurrent response (Figure 6d), which further confirms the synergistic effect caused by amorphous Ti(IV) and MQDs co-catalysts co-deposited on the ZIS nanosheets surface; thus, the strongest photocurrent was produced. Meanwhile, an electron paramagnetic resonance (EPR) test was used to explore the formation of active free radicals in the photocatalytic reaction of ZIS, ZIS/Ti(IV), MQDs/ZIS, and MQDs/ZIS/Ti(IV) samples to explore the separation of photogenerated electron-hole pairs. We used 2,2,6,6-tetramethylpiperidinooxy (TEMPO) and 5,5-dimethyl-1-pyrroline (DMPO) as h^+^ and ·O_2_^−^ trapping agents, respectively. As shown in Figure 7a,b, MQDs/ZIS/Ti(IV) exhibited the weakest TEMPO peak and the strongest DMPO-·O_2_^−^ peak after 5 min of visible light illumination, and the TEMPO and DMPO-·O_2_^−^ peaks of ZIS modified with MQDs or amorphous Ti(IV) alone were between pure ZIS and ZIS co-modified with MQDs and amorphous Ti(IV). The above results demonstrate that the synergistic effect caused by the simultaneous incorporation of amorphous Ti(IV) and MQDs co-catalysts is favorable for the transport of photogenerated carriers, leading to a better photocatalytic H_2_-evolution performance.

### 3.3. Mechanism Research

To clarify the migration of photogenerated electrons over MQDs/ZIS/Ti(IV) in detail, the band structure of MQDs/ZIS/Ti(IV) was determined by DRS and Mott–Schottky (M–S) results. According to the DRS plots, the bandgap energy (E_g_) of pure ZIS and MQDs/ZIS/Ti(IV) was determined to be 2.59 eV and 2.55 eV (Figure 7c). As shown in Figure 7d, the feature of the curve indicates that ZIS is an *n*-type semiconductor. According to the x-intercept of the tangent of the M-S curve, the derived values of the flat-band potential (E_f_) of pure ZIS and ZIS in MQDs/ZIS/Ti(IV) were about −1.0 eV and −0.93 eV (vs. Ag/AgCl, pH = 7), respectively. The reversible hydrogen electrode potential (RHE) and Ag/AgCl electrode potential can be converted via

$$E\left(\mathrm{RHE}\right)=E\left(\mathrm{Ag}/\mathrm{AgCl}\right){+E}^{\theta }\left(\mathrm{Ag}/\mathrm{AgCl}\right)+0.059\mathrm{pH}$$

$$\left(E^{\theta }\left(\mathrm{Ag}/\mathrm{AgCl}\right)=0.197\;\mathrm{eV},\;\mathrm{pH}=7\right)$$

Thus, the E_f_ values of pure ZIS and ZIS in MQDs/ZIS/Ti(IV) were about −0.39 eV and −0.32 eV (vs. RHE, pH = 7), respectively. It is noteworthy that the E_f_ of ZIS in MQDs/ZIS/Ti(IV) was more positive than that of pure ZIS, indicating that the E_f_ of ZIS decreased after the combination of ZIS and MQDs. This result suggests that the close contact between ZIS and MQDs in the MQDs/ZIS/Ti(IV) complex led to the transfer of photogenerated electrons from the conduction band (CB) of ZIS to MQDs, namely, a Schottky junction is formed at the ZIS-MQDs interface to balance the E_f_ between the two materials (according to previous reports [38,57], the E_f_ of MQDs is about −0.11 eV (vs. RHE, pH = 7)). During the equilibrium process, the band structure of ZIS “bent upward” due to the formation of a space charge layer on the ZIS side of the ZIS-MQDs interface. However, the ultra-thin nanosheet structure of ZIS microspheres limits the upward bending range of its energy band structure, so the photogenerated electrons on the CB of ZIS can still migrate to the E_f_ of MQDs after the CB is “bent upward” [35,38,39,41]. Generally, the conduction band (CB) potentials of the *n*-type semiconductors were about 0.2 eV higher than the E_f_, thus the CB of ZIS in MQDs/ZIS/Ti(IV) was about −0.52 eV (vs. RHE, pH = 7). Correspondingly, the valence band (VB) potentials were calculated $\left(E_{\mathrm{CB}}{=E}_{\mathrm{VB}}{-E}_{g}\right)$ as 2.03 eV.

**Figure 8:**
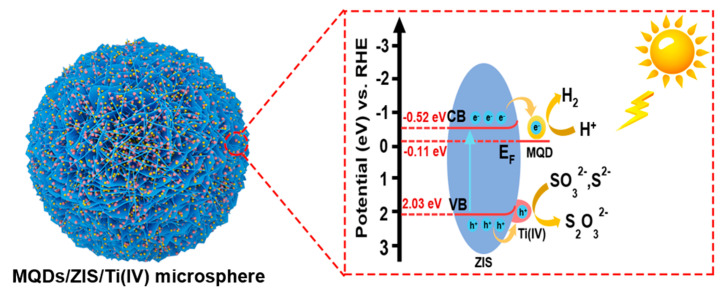
The mechanism of photocatalytic H_2_-evolution on the MQDs/ZIS/Ti(IV) composite photocatalyst.

Based on the above results and discussions, a possible mechanism for the high photocatalytic H_2_-evolution activity of the novel flower-like ZIS microsphere photocatalyst co-modified by amorphous Ti(IV) and MQDs co-catalysts was proposed (Figure 8). Under the irradiation of simulated sunlight, electrons are photoexcited from the VB of ZIS in MQDs/ZIS/Ti(IV) to its CB, and photogenerated holes stay on the VB of ZIS in MQDs/ZIS/Ti(IV). The ZIS-MQDs interface forms a Schottky junction, and the photogenerated electrons on the CB of ZIS shift to MQDs because the E_f_ of MQDs is close to Pt and more positive than the CB level of ZIS. Since the Schottky barrier also has the advantage of preventing electron backflow, the separation of photogenerated electron-hole pairs is improved more effectively. Moreover, numerous surface terminal functional groups (-OH, C = O, C-F and Ti-O) of MQDs can lead to a strong interface connection with ZIS, and the excellent metal conductivity of MQDs is conducive to the transfer of the electron to its surface, which eventually facilitates a reduction in the water-splitting reaction. At the same time, the presence of amorphous Ti(IV) nanoclusters on the surface of ZIS is beneficial to the rapid transfer of photogenerated holes from VB of ZIS in MQDs/ZIS/Ti(IV) to amorphous Ti(IV) nanoclusters, which not only prevent the photo-corrosion of ZIS originating from the S^2−^ oxidation with photogenerated holes, but also further enhance the separation of the photogenerated electron-hole pairs. Furthermore, the flower-like microsphere structure of ZIS can offer more deposition sites for amorphous Ti(IV) and MQDs co-catalysts and more active sites for photocatalytic H_2_-evolution reactions, further improving its photocatalytic activity.

## 4. Conclusions

In summary, unique 3D flower-like ZIS microspheres provide numerous attachment sites for the in situ growth of amorphous Ti(IV) and MQDs co-catalysts. They are especially conducive to the transfer of photogenerated carriers between amorphous Ti(IV), ZIS, and MQDs. The optimal MQDs/ZIS/Ti(IV) shows an excellent photocatalytic H_2_-evolution activity of 7.52 mmol·g^−1^·h^−1^ without metallic Pt as the co-catalyst in the presence of Na_2_S/Na_2_SO_3_ as the hole scavenger. The highly efficient photocatalytic H_2_-evolution activity is attributed to the synergistic effect caused by the flower-like microsphere structure, the amorphous Ti(IV) co-catalyst, and the Schottky junction formed at the ZIS-MQDs interface, which can accelerate the separation of photogenerated electron-hole pairs. Therefore, MQDs with excellent metal conductivity, hydrophilicity, and quantum confinement effect, as well as unique physical optical properties, can form a Schottky junction with ZIS and then act as active sites for a reduction in the water-splitting reaction, and amorphous Ti(IV) nanoclusters can act as hole co-catalyst to prevent the photo-corrosion of ZIS from the S^2−^ oxidation induced by photogenerated holes, ensuring the long-term photo-stability of the sample. This work may not only open new avenues for the design of semiconductors with a superficial induction effect to obtain high-efficiency photocatalysts, but also provide more possibilities for the practical application of photocatalytic technology.

## Supplementary Materials

The following supporting information can be downloaded at: https://www.mdpi.com/article/10.3390/nano12030542/s1, Figure S1: SEM images (a,b) and corresponding element (S, In and Zn) mappings (c) of ZIS, Figure S2: SEM images (a,b) and corresponding element (S, In, Zn and Ti) mappings (c) of ZIS/Ti(IV), Figure S3: N_2_ adsorption−desorption isotherm curves of ZIS, ZIS/Ti(IV), MQDs/ZIS, MQDs/ZIS/Ti(IV), and MNSs/ZIS/Ti(IV), Figure S4: TEM images of MQDs (a) and enlarged portion (b) of MQDs TEM images, Figure S5: Diameter distribution of MQDs, Figure S6: S 2p (a), In 3d (b), Zn 2p (c), and Ti 2p (d) XPS spectra of ZIS and ZIS/Ti(IV), Figure S7: Histogram of average H_2_ production rates of ZIS, ZIS/Ti(IV), MQDs/ZIS, and MQDs/ZIS/Ti(IV) and the sum of ZIS/Ti(IV) and MQDs/ZIS, Figure S8a–d: GC diagram of the best sample (MQDs/ZIS/Ti(IV)) at each time (1, 2, 3, and 4 h), Figure S9: SEM image of MNSs, Figure S10: FT-IR spectra of MQDs and MNSs, Figure S11: SEM images (a,b) and corresponding element (S, In, Zn, Ti and C) mappings (c) of MNSs/ZIS/Ti(IV), Figure S12: UV–vis diffuse reflectance spectra of MQDs/ZIS/Ti(IV), MNSs/ZIS/Ti(IV), MQDs, and MNSs samples, Figure S13: XRD patterns of MQDs/ZIS/Ti(IV) fresh and after photocatalytic H_2_ evolution test, Figure S14: XPS spectra of MQDs/ZIS/Ti(IV) fresh and after photocatalytic H_2_ evolution test: survey (a), S 2p (b), In 3d (c), Zn 2p (d), Ti 2p (e), and C 1s (f), Table S1: Photocatalytic H_2_ evolution of ZIS, ZIS/Ti(IV), MQDs_(x)_/ZIS, and MQDs_(x)_/ZIS/Ti(IV) samples under simulated sunlight irradiation (AM 1.5), Table S2: ICP-MS test results of ZIS/Ti(IV) and MQDs/ZIS/Ti(IV) samples, Table S3: Photocatalytic H_2_ evolution compared with other works on ZIS photocatalysts integrated with quantum dots, Table S4: Photocatalytic H_2_ evolution of MQDs/ZIS/Ti(IV) and MNSs_(x)_/ZIS/Ti(IV) samples under simulated sunlight irradiation (AM. 1.5), Table S5: Exponential decay-fitted parameters of the fluorescence lifetimes of ZIS, ZIS/Ti(IV), MQDs/ZIS, sMQDs/ZIS/Ti(IV), and MNSs/ZIS/Ti(IV); Table S6: The exponential decay-fitted parameters of the fluorescence lifetime of ZIS, ZIS/Ti(IV), MQDs/ZIS, MQDs/ZIS/Ti(IV) and MNSs/ZIS/Ti(IV).
