# Supplementary material for: High-Stability Ti3C2-QDs/ZnIn2S4/Ti(IV) Flower-like Heterojunction for Boosted Photocatalytic Hydrogen Evolution
Source: Nanomaterials (Basel). 2022 Feb 5;12(3):542. doi: 10.3390/nano12030542 (PMC8840382; doi:10.3390/nano12030542)
Supplement: Supplementary file 1 [file nanomaterials-12-00542-s001.zip › nanomaterials-1525715-supplementary.pdf]

nanomaterials

Supporting Information

High-Stability Ti_3C_2-QDs/$ZnIn_2S_4$/Ti(IV) Flower-Like Heterojunction for Boosted Photocatalytic Hydrogen Evolution

Liqin Yang [1], Zhihong Chen [2,3], Xin Wang [1,2] and Mingliang Jin [1,2,*]

[1] National Center for International Research on Green Optoelectronics, South China Academy of Advanced Optoelectronics, South China Normal University, Guangzhou 510006, China; yangliqin2019@126.com (L.Y.); wangxin@scnu.edu.cn (X.W.)
[2] International Academy of Optoelectronics at Zhaoqing, South China Normal University, Zhaoqing 526000, China; chenzhihong1227@sina.com
[3] Key Laboratory for Water Quality and Conservation of the Pearl River Delta, Ministry of Education, Institute of Environmental Research at Greater Bay, Guangzhou University, Guangzhou 510006, China
* Correspondence: jinml@scnu.edu.cn

1. Experimental

1.1. Characterization

The morphology and structure of samples were observed by field-emission scanning electron microscopy (FESEM, ZEISS Gemini 500, Carl Zeiss, Germany) and transmission electron microscopy (TEM, JEM-2100HR, Tokyo, Japan). X-ray powder diffraction (XRD) and Fourier-transform infrared (FT-IR) patterns of the as-prepared samples were conducted on a Bruker D8 Advance X-ray diffractometer (Karlsruhe, Germany) with Cu Kα X-ray source and a Thermo spectrometer, respectively, to expound the crystalline and chemical structure. The inductively coupled optical emission spectrometry (ICP-MS, Agilent 7800, Upland, CA, USA) was performed to analyze the composition of the samples. The chemical and valence states of involved elements were carried out by X-ray photoelectron spectroscopy (XPS, Thermo Fisher Scientific ESCALAB Xi^+, Waltham, MA, USA) with a monochromatic Al K-alpha radiation source. UV-vis diffuse reflectance spectroscopy (DRS) was measured from a UV-vis spectrophotometer (HITACHI U-41000, Tokyo, Japan) to obtain the optical property of the as-prepared samples.

The steady-state photoluminescence (PL) spectra were carried out on a fluorescence spectrometer (Hitachi F-4600, Tianjin, China). Time-resolved photoluminescence (TR-PL) spectra were recorded with a single photon counting spectrometer from Horiba Instrument, Fluorolog-3, Irvine, CA, USA. The time resolution was determined to be ps level from the instrument response function (IRF), and the TR-PL decay effective lifetimes (τ_{eff}) of the sample is at ns level, so the influence of the instrument on the experimental results is negligible. In the TR-PL decay spectra measurement, the measured TR-PL decay curve is the convolution of the IRF and the ideal time decay curve, and the IRF has been deducted by the built-in software of the instrument. The specific surface area (BET) was examined by the N_2 adsorption-desorption experiments (Quantachrome Instruments, Shanghai, China). The electron paramagnetic resonance (EPR) spectra of liquid samples were recorded on a Brukera 300 spectrometer, Brooke, Germany.

1.2. Photoelectrochemical Measurements

The photoelectrochemical tests of the as-prepared samples were performed on an electrochemical workstation (Shanghai, CHI660E, China) to explore the charge separation. In the process of testing, the prepared photocatalyst, Pt sheet and Ag/AgCl electrode (3 M KCl) were used as working, counter and reference electrodes, respectively. Typically, 5.0 mg photocatalyst and 10 μL Nafion (5 wt%) solution were added into the mixture of 400 μL water and isopropanol (3:1 v/v). After ultrasonic treatment for 60 min, a uniform colloidal solution was obtained. Finally, the colloidal solution was deposited on a clean FTO

with an area of 1 cm^2, and the working electrode was obtained after drying in the natural state. The 0.2 M of Na_2SO_4 aqueous solution and a Xe lamp (300 W) equipping a cut-off filter (AM 1.5) were used as the electrolyte and the light source in the measurements, respectively.

1.3. Photocatalytic H_2 Production

Briefly, 20 mg photocatalysts and 100 mL aqueous solution (0.35 M Na_2S/0.25 M Na_2SO_3) was added to a top-irradiation quartz photoreactor vessel with closed gas circulation and evacuation system and sealed with a rubber septum. Before the photocatalytic reaction, the photoreactor was vacuumed to eliminate air for 60 min and the circulating cooling water was connected to maintain the inside temperature. Then, it was irradiated by a 300-W Xe lamp equipped with a cut-off filter (AM 1.5) to provide the simulated sunlight irradiations (Perfect Light, Beijing, China). The produced H_2 was quantified by Labsolar-6A online photocatalytic analysis system (Perfect Light technology Co.LTD, Beijing, China) and gas chromatography (Techcomp GC7900, Shanghai, China, equipped with a thermal conductivity detector (TCD) and 5 Å molecular sieve columns). The calibration curve method was used for the quantitative analysis of H_2 production. The procedure of standardizing the calibration curve is as follows: under the identical atmosphere system as photocatalytic H_2 production, a series of different H_2 volume ratios were automatically injected and detected by GC to obtain a series of points for calibration (H_2 volume is used as abscissa and H_2 peak area in GC pattern is used as ordinate). Each calibration curve point is required to fall on the calibration curve as much as possible, and the correlation coefficient r^2 of the curve is greater than 0.999, ensuring the accuracy and linearity of the calibration curve. The raw data of points (H_2 volume vs H_2 peak area in GC pattern) was listed as follows:

**H_2 volume (mL)**	**0.5**	**1**	**2**	**3**	**4**
H_2 peak area in GC pattern (uV*S)	47,621	112,309	230,679	351,619	464,235

Finally, the H_2 evolution quantitative calibration curve in this work has been deduced as follows: $y = 118988 * x - 8582.4, r^2 = 0.9996$ (y refers to the H_2 peak area in GC pattern (uV*S), x refers to H_2 volume (mL), r^2 refers to correlation coefficient). The gas volume of each injection is 1 mL, and the activities of photocatalysts are compared using the average H_2 evolution rate in the first 4 h.

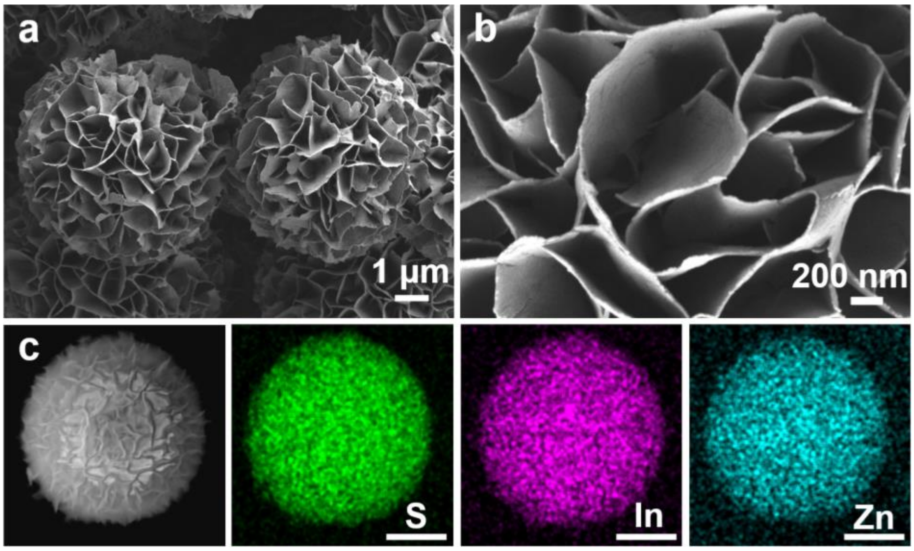

Figure S1. SEM images (**a**,**b**) and corresponding element (S, In and Zn) mappings (**c**) of ZIS.

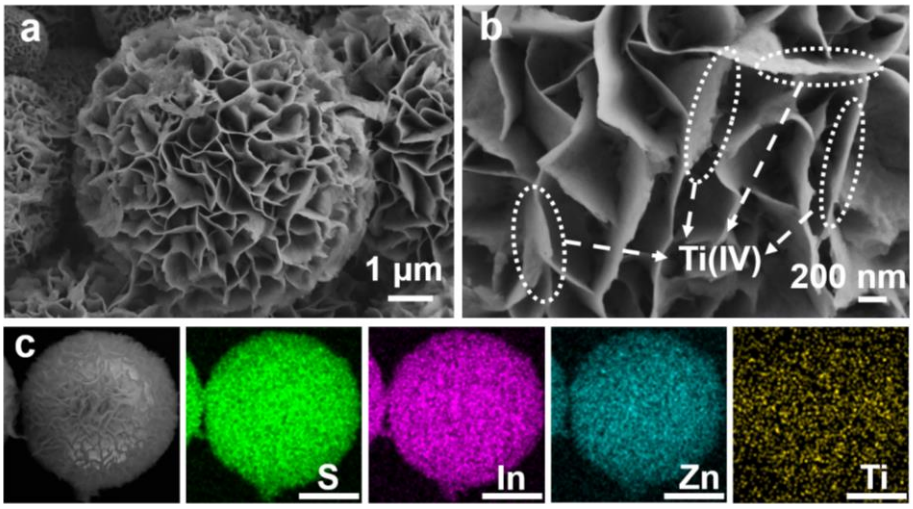

Figure S2. SEM images (**a**,**b**) and corresponding element (S, In, Zn and Ti) mappings (**c**) of ZIS/Ti(IV).

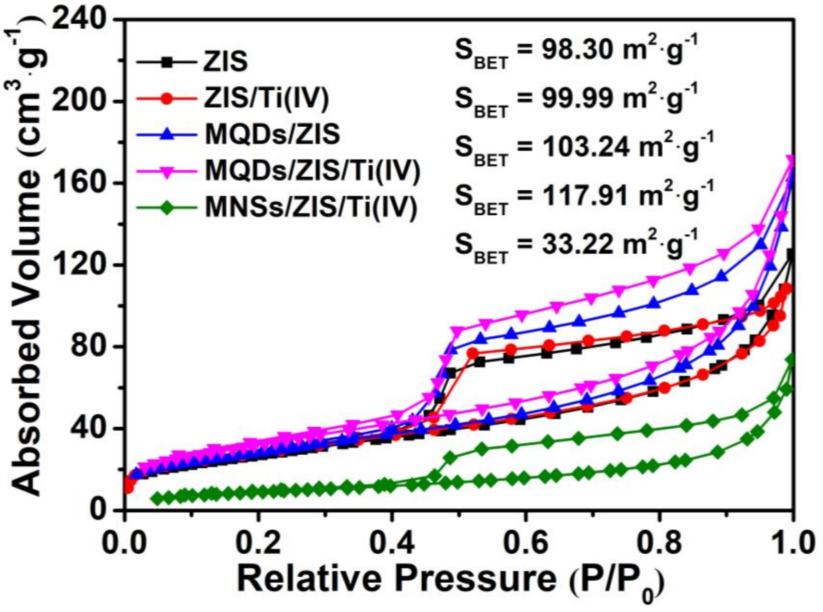

Figure S3. N_2 adsorption−desorption isotherms curves of ZIS, ZIS/Ti(IV), MQDs/ZIS, MQDs/ZIS/Ti(IV) and MNSs/ZIS/Ti(IV).

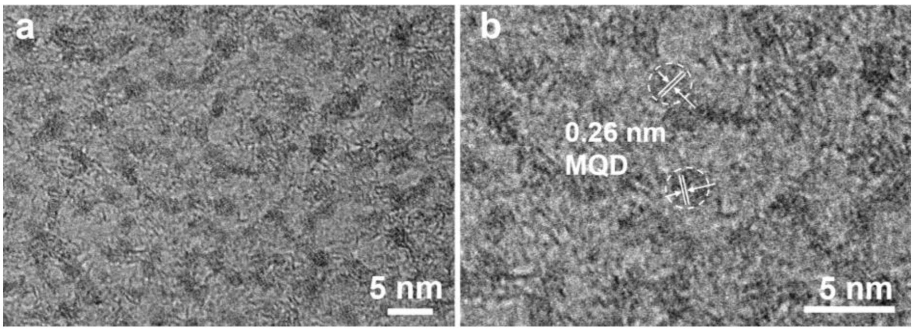

Figure S4. TEM images of MQDs (a) and enlarged portion (b) of MQDs TEM images.

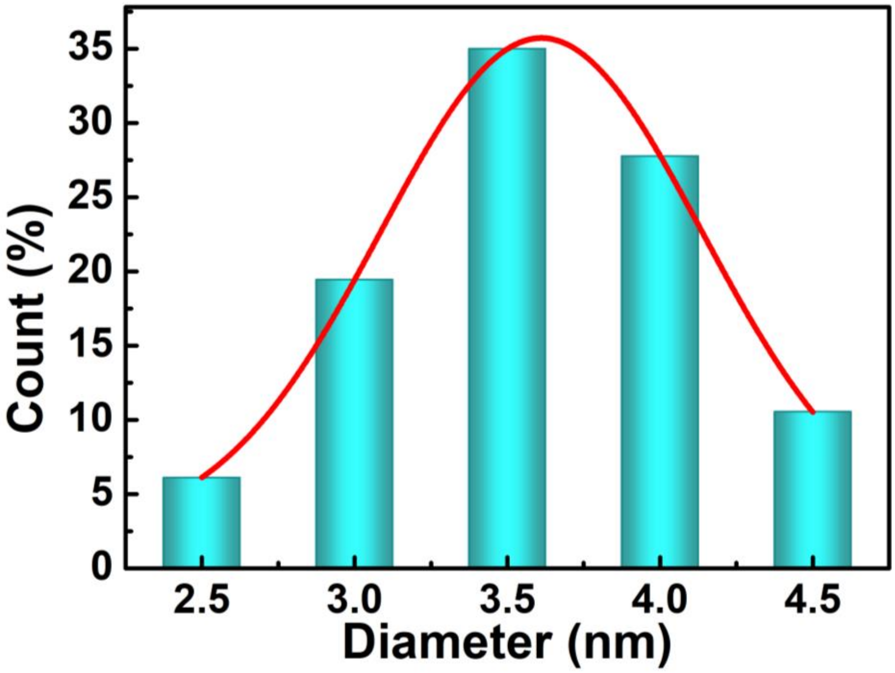

Figure S5. The diameter distribution of MQDs.

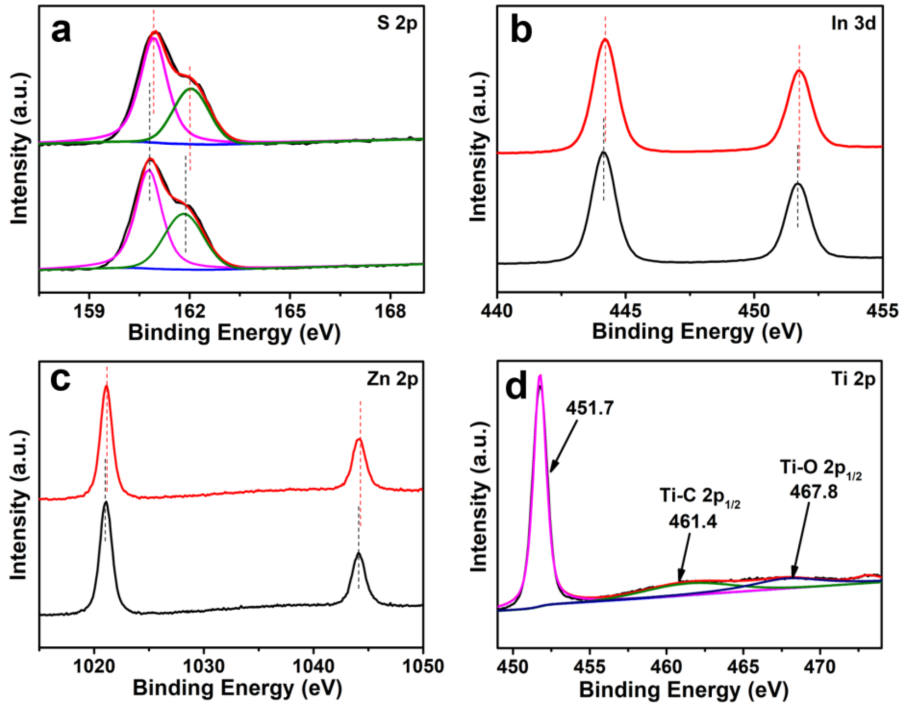

Figure S6. S 2p (**a**), In 3d (**b**), Zn 2p (**c**) and Ti 2p (**d**) XPS spectra of ZIS and ZIS/Ti(IV).

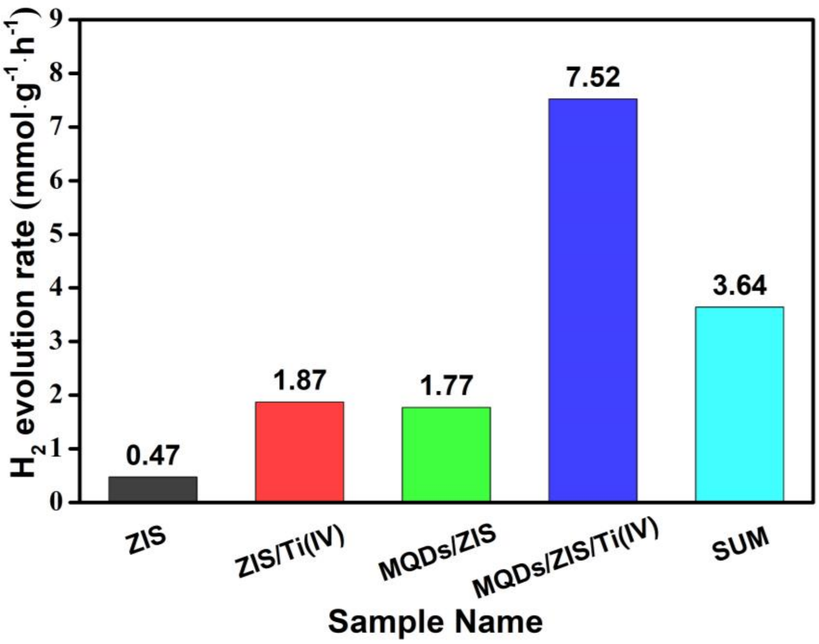

Figure S7. Histogram of average H_2 production rates of ZIS, ZIS/Ti(IV), MQDs/ZIS, MQDs/ZIS/Ti(IV) and the sum of ZIS/Ti(IV) and MQDs/ZIS.

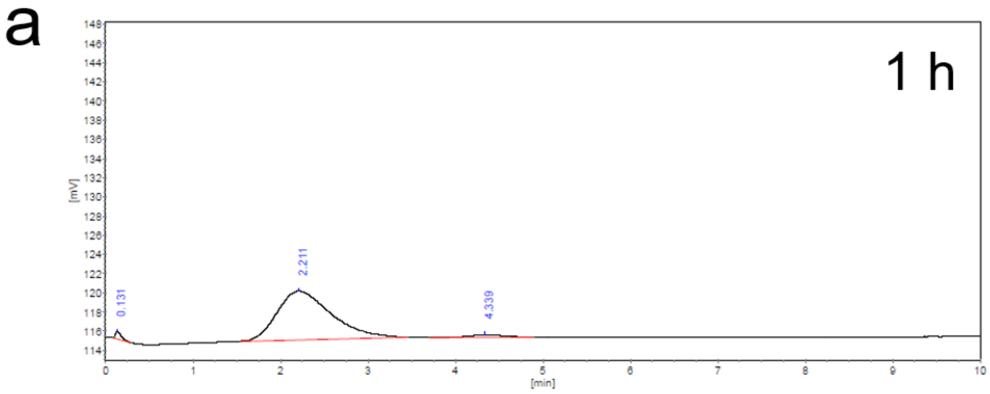

Analysis Results

No.	Compound Name	R.Time [min]	Height [uV]	Area [uV*s]	Area%	Conc. [mg/mL]	Type
1		0.131	754	3780	1.6259	0.0000	BB
2	H2	2.211	5071	219766	94.5199	0.0000	BB
3		4.339	273	8961	3.8542	0.0000	BB
	**Total:**		**6098**	**232507**	**100.0000**	**0.0000**	

b

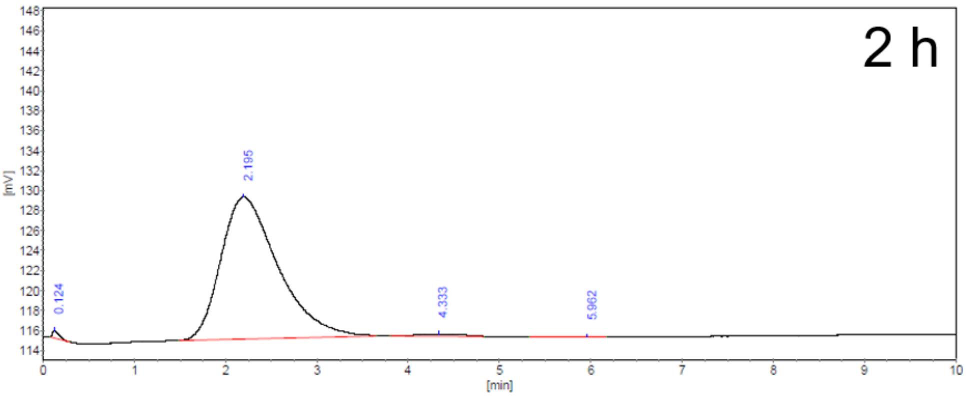

Analysis Results

No.	Compound Name	R.Time [min]	Height [uV]	Area [uV*s]	Area%	Conc. [mg/mL]	Type
1		0.124	751	3697	0.5874	0.0000	BB
2	H2	2.195	14158	616600	97.9556	0.0000	BB
3		4.333	241	7680	1.2201	0.0000	BB
4		5.962	32	1179	0.1873	0.0000	BB
5		10.680	29	313	0.0497	0.0000	BB
	**Total:**		**15211**	**629469**	**100.0000**	**0.0000**	

C

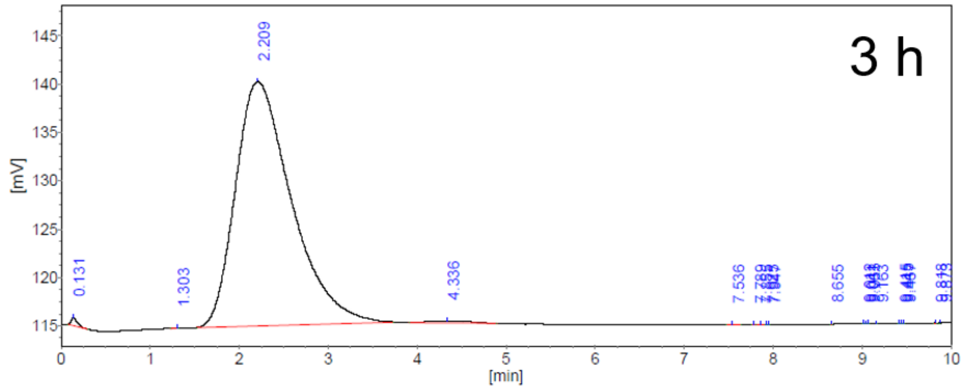

Analysis Results

No.	Compound Name	R.Time [min]	Height [uV]	Area [uV*s]	Area%	Conc. [mg/mL]	Type
1		0.131	783	3908	0.3502	0.0000	BB
2		1.303	29	91	0.0082	0.0000	BB
3	H2	2.209	25239	1104034	98.9229	0.0000	BB
4		4.336	252	7384	0.6616	0.0000	BB
5		7.536	27	46	0.0041	0.0000	BB
6		7.789	32	59	0.0052	0.0000	BV
7		7.855	32	177	0.0159	0.0000	VV
8		7.925	32	39	0.0035	0.0000	VV
9		7.947	32	16	0.0015	0.0000	VB
10		8.655	14	7	0.0006	0.0000	BB
11		9.012	32	37	0.0033	0.0000	BV
12		9.041	22	14	0.0012	0.0000	VV
13		9.063	32	19	0.0017	0.0000	VB
14		9.163	19	10	0.0009	0.0000	BB
15		9.415	22	11	0.0010	0.0000	BB
16		9.445	19	10	0.0009	0.0000	BV
17		9.467	24	12	0.0011	0.0000	VB
18		9.818	32	26	0.0023	0.0000	BB
19		9.873	24	12	0.0011	0.0000	BB
20		10.585	27	14	0.0012	0.0000	BB
21		10.618	32	24	0.0021	0.0000	BV
22		10.640	32	35	0.0031	0.0000	VV
23		10.673	32	71	0.0064	0.0000	VB
	**Total:**		**26821**	**1116055**	**100.0000**	**0.0000**	

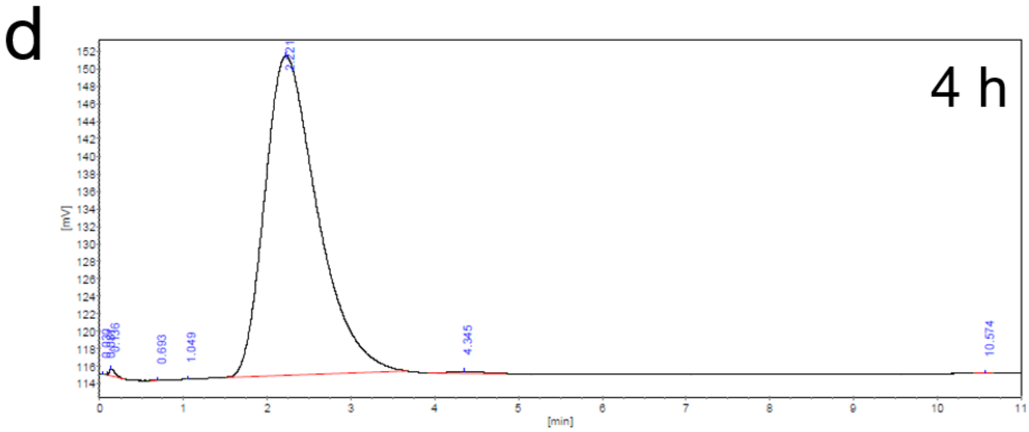

Analysis Results

No.	Compound Name	R.Time [min]	Height [uV]	Area [uV*s]	Area%	Conc. [mg/mL]	Type
1		0.030	19	10	0.0006	0.0000	BB
2		0.081	73	67	0.0042	0.0000	BV
3		0.136	800	4003	0.2494	0.0000	VB
4		0.693	27	104	0.0065	0.0000	BB
5		1.049	15	6	0.0004	0.0000	BB
6	H2	2.221	36463	1594854	99.3533	0.0000	BB
7		4.345	218	5989	0.3731	0.0000	BB
8		10.574	32	203	0.0126	0.0000	BB
	**Total:**		**37647**	**1605236**	**100.0000**	**0.0000**	

Figure S8. a–d. The GC diagram of the best sample (MQDs/ZIS/Ti(IV)) at each time (1, 2, 3 and 4 h).

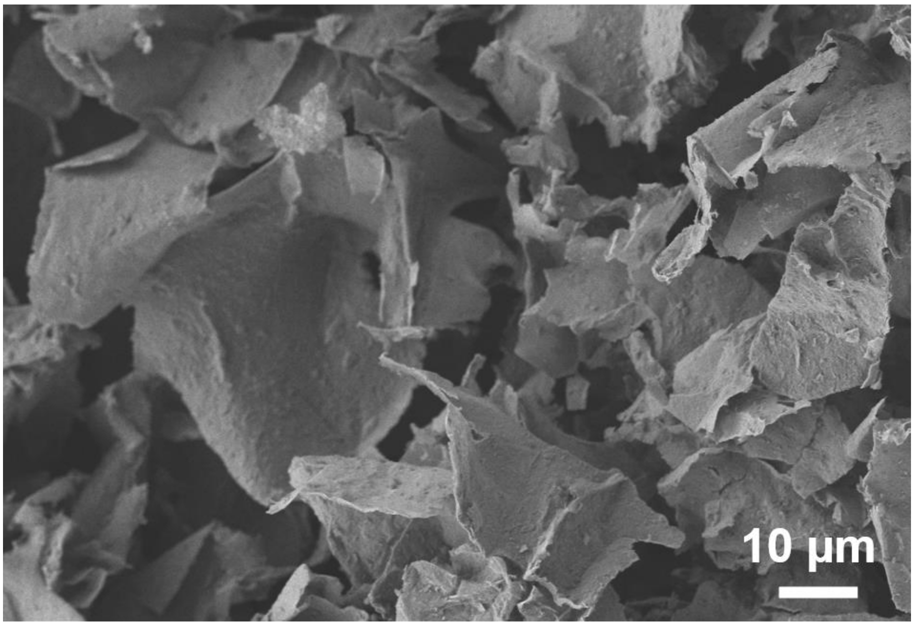

Figure S9. SEM image of MNSs.

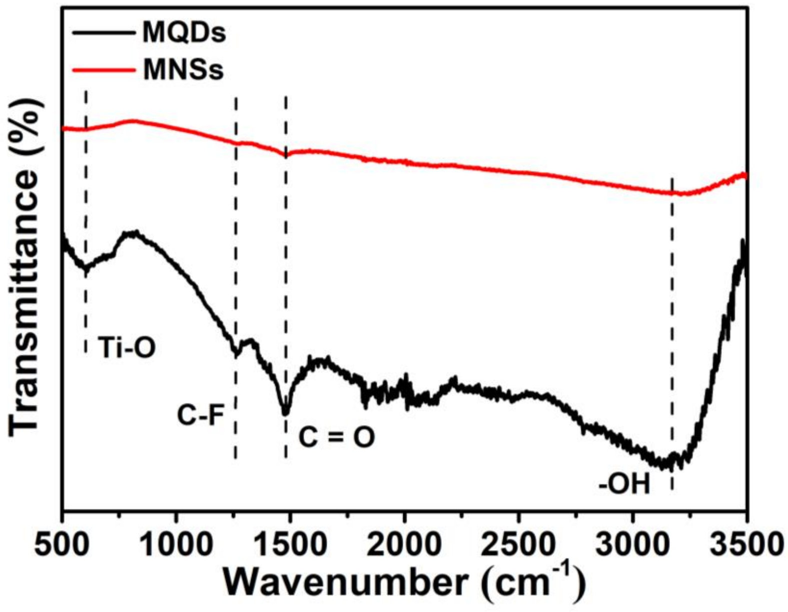

Figure S10. The FT-IR spectra of MQDs and MNSs.

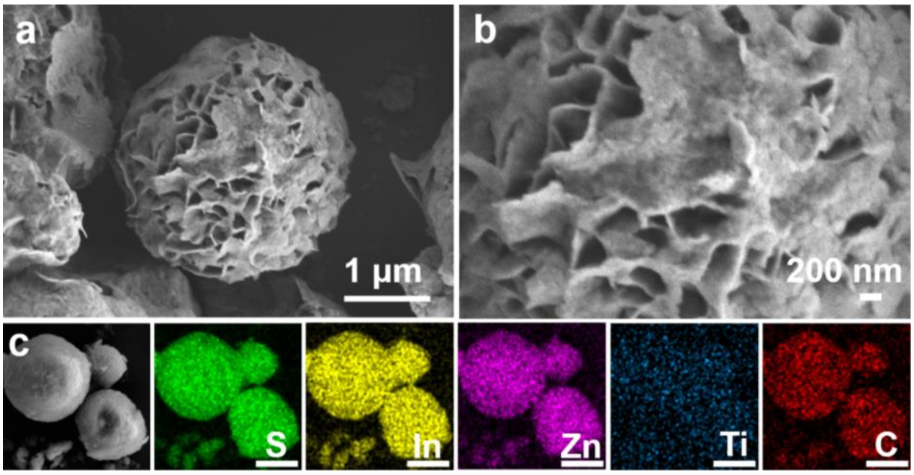

Figure S11. SEM images (**a**,**b**) and corresponding element (S, In, Zn, Ti and C) mappings (**c**) of MNSs/ZIS/Ti(IV).

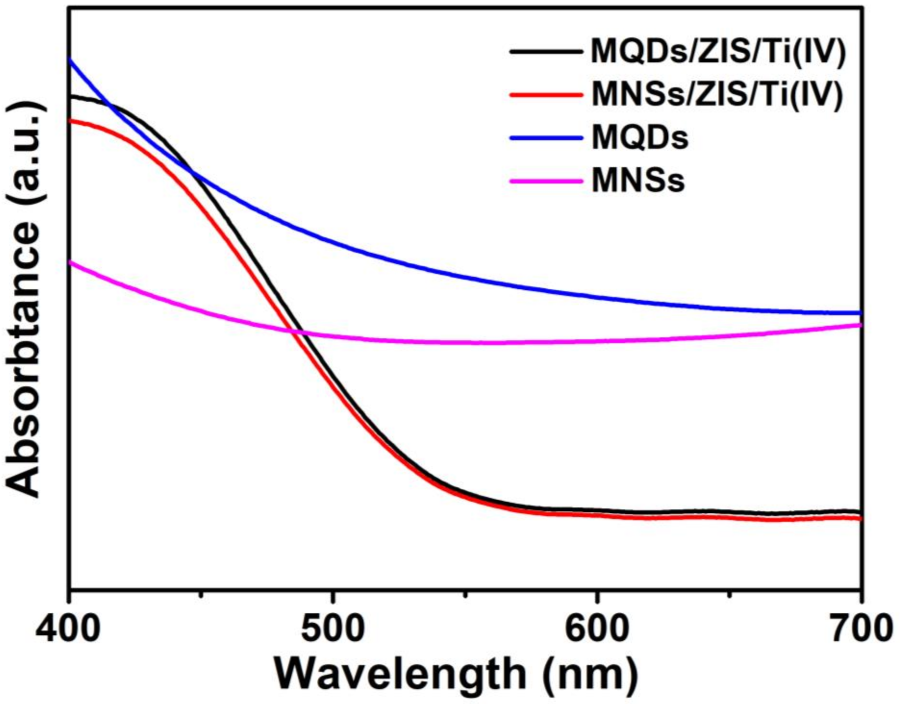

Figure S12. UV–vis diffuse reflectance spectra of MQDs/ZIS/Ti(IV), MNSs/ZIS/Ti(IV), MQDs and MNSs samples.

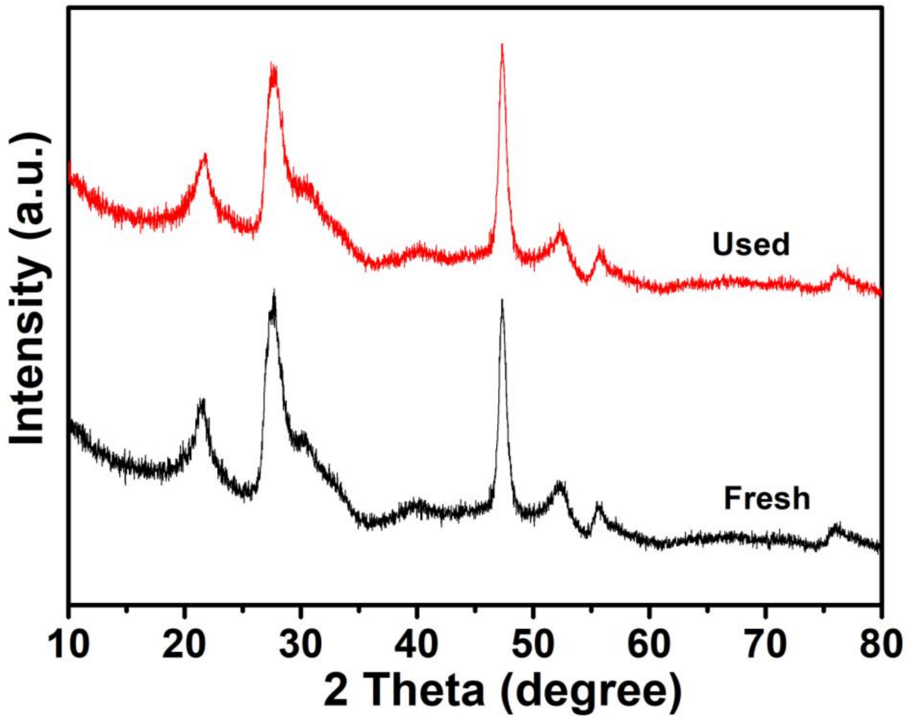

Figure S13. The XRD patterns of MQDs/ZIS/Ti(IV) fresh and after photocatalytic H_2 evolution test.

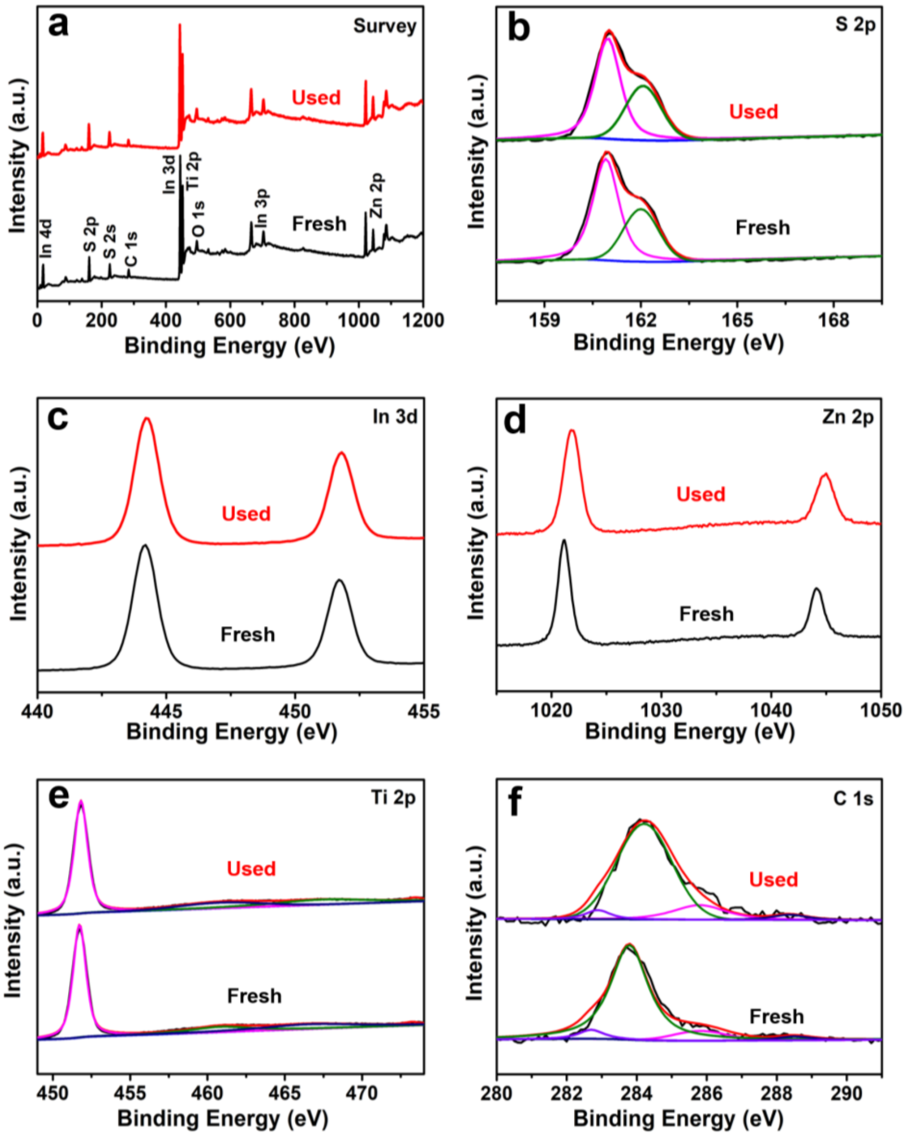

Figure S14. The XPS spectra of MQDs/ZIS/Ti(IV) fresh and after photocatalytic H_2 evolution test: survey (**a**), S 2p (**b**), In 3d (**c**), Zn 2p (**d**), Ti 2p (**e**) and C 1s (**f**).

Table S1. The photocatalytic H_2 evolution of ZIS, ZIS/Ti(IV), $MQDs_{(x)}$/ZIS, and $MQDs_{(x)}$/ZIS/Ti(IV) samples under simulated sunlight irradiation (AM 1.5).

**Samples**	**H_2 Peak Area in GC Pattern (uV*S)**				Quantitative Analysis of Photocatalytic H_2 Production Calibration Curve	Photocatalytic Activity ($mmol\cdot g^{-1}\cdot h^{-1}$)
	**1 h**	**2 h**	**3 h**	**4 h**		
ZIS	19,446	43,741	64,974	92,490	$y = 118988 * x - 8582.4 r^2 = 0.9996$ y refers to the H_2 peak area in GC pattern (uV*S)x refers to H_2 volume (mL)r^2 refers to correlation coefficient	0.47
ZIS/Ti(IV)	81,851	174,693	270,543	390,684		1.87
$MQDs_{(1)}$/ZIS	49,065	106,299	162,497	227,366		1.11
$MQDs_{(2)}$/ZIS	88,811	166,357	273,930	368,341		1.77
$MQDs_{(3)}$/ZIS	69,054	165,730	246,141	368,315		1.77
$MQDs_{(4)}$/ZIS	21,045	82,542	205,524	344,216		1.65
$MQDs_{(1)}$/ZIS/Ti(IV)	163,254	380,614	646,975	923,286		4.37
$MQDs_{(2)}$/ZIS/Ti(IV)	219,766	616,600	1,104,034	1,594,854		7.52
$MQDs_{(3)}$/ZIS/Ti(IV)	97,544	297,011	485,846	691,635		3.28
$MQDs_{(4)}$/ZIS/Ti(IV)	108,934	260,842	471,674	646,490		3.07

NOTE: The 2 wt% MQDs doped ZIS and ZIS/Ti(IV) displays the highest photocatalytic H_2-production activity in MQDs(x)/ZIS and MQDs(x)/ZIS/Ti(IV) samples, respectively. Therefore, the sample with the best photocatalytic activity is selected as the representative to investigate their optical and physicochemical properties. In the text part, MQDs/ZIS and MQDs/ZIS/Ti(IV) are corresponded to $MQDs_{(2)}$/ZIS and $MQDs_{(2)}$/ZIS/Ti(IV), respectively.

Table S2. The ICP-MS test results of ZIS/Ti(IV) and MQDs/ZIS/Ti(IV) samples.

Element	In ($mg{\cdot}g^{-1}$)	Zn ($mg{\cdot}g^{-1}$)	Ti ($mg{\cdot}g^{-1}$)
ZIS/Ti(IV)	458.9	120.9	0.3
MQDs/ZIS/Ti(IV)	415.2	117.2	3.0

Table S3. The photocatalytic H_2 evolution compared with other works on ZIS photocatalysts integrated with quantum dots.

Photocatalysts	Co-catalyst Metal	Photocatalytic Activity ($mmol{\cdot}g^{-1}{\cdot}h^{-1}$)	References
Ti_3C_2 MXene QDs/$ZnIn_2S_4$/Ti(IV)	free	7.52	This work
$CuInS_2$ QDs/$ZnIn_2S_4$	0.75 wt% of Pt	1.04	[S1]
CdTe QDs/$ZnIn_2S_4$	free	0.34	[S2]
CdS/MoS_2 QDs/$ZnIn_2S_4$	free	2.11	[S3]
AgO_2 QDs/$ZnIn_2S_4$	3 wt% of Pt	0.47	[S4]
BPQDs@Cu_7S_4/$ZnIn_2S_4$	free	0.89	[S5]
MoC QDs/C/$ZnIn_2S_4$	free	1.13	[S6]

Table S4. The photocatalytic H_2 evolution of MQDs/ZIS/Ti(IV) and $MNSs_{(x)}$/ZIS/Ti(IV) samples under simulated sunlight irradiation (AM. 1.5).

Samples	H_2 Peak Area in GC Pattern (uV*S)				Quantitative Analysis of Photocatalytic H_2 Production Calibration Curve	Photocatalytic Activity ($mmol{\cdot}g^{-1}{\cdot}h^{-1}$)
	1 h	2 h	3 h	4 h		
MQDs/ZIS/Ti(IV)	219,766	616,600	1,104,034	1,594,854	y = 118988 * x - 8582.4	7.52
$MNSs_{(1)}$/ZIS/Ti(IV)	122,533	243,689	364,789	492,713	r^2 = 0.9996 y refers to the H_2 peak	2.35
$MNSs_{(2)}$/ZIS/Ti(IV)	168,956	328,449	563,897	809,243	area in GC pattern (uV*S)x refers to	3.84
$MNSs_{(3)}$/ZIS/Ti(IV)	125,506	213,609	302,961	392,951	H_2 volume (mL)	1.88
$MNSs_{(4)}$/ZIS/Ti(IV)	103,470	190,106	265,768	343,816	r^2 refers to correlation coefficient	1.65

Table S5. The photocatalyst H_2 evolution of 4 cycling stability tests of MQDs/ZIS/Ti(IV) under simulated sunlight irradiation (AM 1.5).

Cycle Index	H_2 Peak Area in GC Pattern (uV*S)				Quantitative Analysis of Photocatalytic H_2 Production Calibration Curve	Photocatalytic Activity ($mmol{\cdot}g^{-1}{\cdot}h^{-1}$)
	1 h	2 h	3 h	4 h		
1st	219,766	616,600	1,104,034	1,594,854	y = 118988 * x - 8582.4	7.52
2nd	382,210	776,262	1,151,478	1,556,524	r^2 = 0.9996y refers to the H_2 peak area in	7.34
3th	433,124	839,545	1,180,133	1,547,258	GC pattern (uV*S)x refers to H_2 volume	7.30
4th	418,276	808,624	1,175,932	1,499,172	(mL)r^2 refers to correlation coefficient	7.07

Table S6. The exponential decay-fitted parameters of the fluorescence lifetime of ZIS, ZIS/Ti(IV), MQDs/ZIS, MQDs/ZIS/Ti(IV) and MNSs/ZIS/Ti(IV).

Samples	A_1	τ_1	A_2	τ_2	τ_{eff}
ZIS	0.50312	0.4327	0.28178	11.56616	4.43

ZIS/Ti(IV)	0.85249	7.42244	0.85248	7.42151	7.42
MQDs/ZIS	0.49565	2.60739	0.09761	29.86587	7.09
MQDs/ZIS/Ti(IV)	0.17794	52.17659	0.25204	5.56178	24.85
MNSs/ZIS/Ti(IV)	0.1828	47.30022	1.07879	3.43148	9.79

References

1. Cavdar, O.; Malankowska, A.; Amgar, D.; Mazierski, P.; Łuczak, J.; Lisowski, W.; Zaleska-Medynska, A. Remarkable visible-light induced hydrogen generation with ZnIn2S4 microspheres/CuInS2 quantum dots photocatalytic system. *International Journal of Hydrogen Energy* **2021**, *46*, 486–498.
2. Janani, R.; Sumathi, S.; Gupta, B.; Shaheer, A.R.M.; Ganapathy, S.; Neppolian, B.; Roy, S.C.; Channakrishnappa, R.; Paul, B.; Singh, S. Development of CdTe quantum dot supported ZnIn2S4 hierarchical microflowers for improved photocatalytic activity. *J. Environ. Chem. Eng.* **2022**, *10*, 107030.
3. Chen, W.; Yan, R.-Q.; Zhu, J.-Q.; Huang, G.-B.; Chen, Z. Highly efficient visible-light-driven photocatalytic hydrogen evolution by all-solid-state Z-scheme CdS/QDs/ZnIn2S4 architectures with MoS2 quantum dots as solid-state electron mediator. *Appl. Surf. Sci.* **2020**, *504*, 144406.
4. Xiao, Y.; Peng, Z.; Zhang, W.; Jiang, Y.; Ni, L. Self-assembly of Ag2O quantum dots on the surface of ZnIn2S4 nanosheets to fabricate p-n heterojunctions with wonderful bifunctional photocatalytic performance. *Appl. Surf. Sci*. **2019**, *494*, 519–531.
5. Zhang, Q.; Zhang, J.; Zhang, L.; Yang, F.; Li, L.; Dai, W.-L. Black phosphorus quantum dots facilitate carrier separation for enhancing hydrogen production over hierarchical Cu7S4/ZnIn2S4 composites. *Catal. Sci. Technol.* **2020**, *10*, 1030–1039.
6. Gao, F.; Zhao, Y.; Zhang, L.; Wang, B.; Wang, Y.; Huang, X.; Wang, K.; Feng, W.; Liu, A.P. Well Dispersed MoC Quantum Dots in Ultrathin Carbon Film as Efficient Co-catalyst for Photocatalytic H2 Evolution. *J. Mater. Chem. A* **2018**, *6*, 18979–18986.
